# parSMURF, a high-performance computing tool for the genome-wide detection of pathogenic variants

**DOI:** 10.1093/gigascience/giaa052

**Published:** 2020-05-23

**Authors:** Alessandro Petrini, Marco Mesiti, Max Schubach, Marco Frasca, Daniel Danis, Matteo Re, Giuliano Grossi, Luca Cappelletti, Tiziana Castrignanò, Peter N Robinson, Giorgio Valentini

**Affiliations:** 1 Università degli Studi di Milano, AnacletoLab - Dipartimento di Informatica, via Giovanni Celoria 18, 20135 Milano, Italy; 2 Berlin Institute of Health (BIH), Anna-Louisa-Karsch-Straße 2, 10178 Berlin, Germany; 3 Charité – Universitätsmedizin Berlin, Chariteplatz 1, 10117 Berlin, Germany; 4 The Jackson Laboratory for Genomic Medicine, 10 Discovery Drive, Farmington (CT) - 06032, United States of America; 5 CINECA, SCAI SuperComputing Applications and Innovation Department, Via dei Tizii 6, 00185 Roma, Italy; 6 University of Tuscia, Department of Ecological and Biological Sciences (DEB), Largo dell'Università snc, 01100 Viterbo, Italy; 7 CINI National Laboratory in Artificial Intelligence and Intelligent Systems - AIIS, Università di Roma, Via Ariosto 25, 00185 Roma, Italy

**Keywords:** high-performance computing tool for genomic medicine, parallel machine learning tool for big data, parallel machine learning tool for imbalanced data, ensemble methods, machine learning for genomic medicine, machine learning for imbalanced genomic data, prediction of deleterious or pathogenic variants, high-performance computing, Mendelian diseases, GWAS

## Abstract

**Background:**

Several prediction problems in computational biology and genomic medicine are characterized by both big data as well as a high imbalance between examples to be learned, whereby positive examples can represent a tiny minority with respect to negative examples. For instance, deleterious or pathogenic variants are overwhelmed by the sea of neutral variants in the non-coding regions of the genome: thus, the prediction of deleterious variants is a challenging, highly imbalanced classification problem, and classical prediction tools fail to detect the rare pathogenic examples among the huge amount of neutral variants or undergo severe restrictions in managing big genomic data.

**Results:**

To overcome these limitations we propose parSMURF, a method that adopts a hyper-ensemble approach and oversampling and undersampling techniques to deal with imbalanced data, and parallel computational techniques to both manage big genomic data and substantially speed up the computation. The synergy between Bayesian optimization techniques and the parallel nature of parSMURF enables efficient and user-friendly automatic tuning of the hyper-parameters of the algorithm, and allows specific learning problems in genomic medicine to be easily fit. Moreover, by using MPI parallel and machine learning ensemble techniques, parSMURF can manage big data by partitioning them across the nodes of a high-performance computing cluster. Results with synthetic data and with single-nucleotide variants associated with Mendelian diseases and with genome-wide association study hits in the non-coding regions of the human genome, involhing millions of examples, show that parSMURF achieves state-of-the-art results and an 80-fold speed-up with respect to the sequential version.

**Conclusions:**

parSMURF is a parallel machine learning tool that can be trained to learn different genomic problems, and its multiple levels of parallelization and high scalability allow us to efficiently fit problems characterized by big and imbalanced genomic data. The C++ OpenMP multi-core version tailored to a single workstation and the C++ MPI/OpenMP hybrid multi-core and multi-node parSMURF version tailored to a High Performance Computing cluster are both available at https://github.com/AnacletoLAB/parSMURF.

## Background

High-throughput biotechnologies, and the development of artificial intelligence methods and techniques, have opened up new research avenues in the context of genomic and personalized medicine [1,2]. In particular machine learning [3], whole-genome sequencing technologies [4,5], and large population genome sequencing projects [6,7] play a central role for the detection of rare and common variants associated with genetic diseases and cancer [8,9].

In this context, while disease-associated variants falling in the protein-coding regions of the genome have been widely studied [10–12], this is not the case for disease-associated variants located in the non-coding regions of the genome, where our understanding of their impact on *cis*- and *trans*-regulation is largely incomplete. Nevertheless, several studies found that most of the potential pathogenic variants lie in the non-coding regions of the human genome [13].

Driven by the aforementioned motivations many efforts have been devoted in recent years by the scientific community to developing reliable tools for the identification and prioritization of “relevant” non-coding genetic variants. CADD is one of the first machine learning–based methods applied for this purpose on a genome-wide scale [14]. By combining different annotations into a single measure for each variant using first an ensemble of support vector machines and in the current version a fast and efficient logistic regression classifier, CADD likely represents the most used and well-known tool to predict deleterious variants [15].

Starting from this work other machine learning–based methods for the detection of deleterious or pathogenic variants have been proposed, ranging from multiple kernel learning techniques [16] to deep neural networks [17,18], multiple kernel learning integrative approaches [16], unsupervised learning techniques to deal with the scarcity of available annotations [19], and linear models for functional genomic data combined with probabilistic models of molecular evolution [20]. Other approaches predicted the effect of regulatory variation directly from sequence using gkm-SVM [21] or deep learning techniques [22]. More details are covered in 2 recent reviews on machine learning methods for the prediction of disease risk in non-coding regions of the human genome [23, 24].

All these tools are faced with relevant challenges related to the rarity of non-coding pathogenic mutations. Indeed neutral variants largely outnumber the pathogenic ones. As a consequence the resulting classification problem is largely unbalanced toward the majority class and in this setting it is wellknown that imbalance-unaware machine learning methods fail to detect examples of the minority class (i.e., pathogenic variants) [25]. Recently several methods showed that by adopting imbalance-aware techniques we can significantly improve predictions of pathogenic variants in non-coding regions. The first one (GWAVA) applied a modified random forest [26], where its decision trees are trained on artificially balanced data, thus reducing the imbalance of the data [27]. A second one (NCBoost) used gradient tree boosting learning machines with partially balanced data, achieving very competitive results in the prioritization of pathogenic Mendelian variants, even if the comparison with the other state-of-the-art methods has been performed without retraining them, but using only their pre-computed scores [28]. The unbalancing issue has been fully addressed by ReMM [29] and hyperSMURF [30], through the application of subsampling techniques to the “negative” neutral variants, and oversampling algorithms to the set of “positive” pathogenic variants. Moreover a large coverage of the training data and an improvement of the accuracy and the diversity of the base learners is obtained through a partition of the training set and a hyper-ensemble approach, i.e., an ensemble of random forests that in turn are ensembles of decision trees. hyperSMURF achieved excellent results in the detection of pathogenic variants in the non-coding DNA, showing that imbalance-aware techniques play a central role to improve predictions of machine learning methods in this challenging task.

Nevertheless these imbalance-aware methods have been usually implemented with no or very limited use of parallel computation techniques, thus making problematic their application to the analysis of big genomic data. Furthermore, the hyperSMURF method is computationally inten.sive and characterized by a large number of learning parameters that need to be tuned to ensure optimal performance, thus requiring prohibitive computing costs, especially with big genomic data.

To address the aforementioned limitations, in this article we propose parSMURF, a novel parallel approach based on hyperSMURF. While other methods suitable for the assessment of the impact of variants located in protein-coding regions are able to run in parallel environments [31], this is not true for the assessment of non-coding variants. The main goal in the design and development of parSMURF is to make available to the scientific community a general and flexible tool for genomic prediction problems characterized by big and/or highly imbalanced data while ensuring state-of-the-art prediction performance. The high computational burden resulting from the proper tuning and selection of the learning (hyper)-parameters is addressed through a scalable and parallel learning algorithm that leverages different levels of parallelization, and a Bayesian optimizer (BO) for their automatic and efficient tuning.

In the remainder we present the parSMURF algorithm, its relationships with its sequential version hyperSMURF, and its 2 different implementations, respectively, for multi-core workstations and for a highly parallel high-performance computing cluster.

In the Results section experiments with big synthetic and actual genomic data show that parSMURF scales nicely with big data and substantially improves the speed-up of the computation. Finally experiments with Mendelian data and genome-wide association studies (GWAS) hits at whole-genome level show that parSMURF considerably outperforms its sequential counterpart hyperSMURF, by exploiting its multiple levels of parallelism and the automatic tuning of its learning hyper-parameters through a grid search or a Bayesian optimization method. parSMURF^1^ multi-thread and hybrid multi-thread and multi-process MPI C++ parSMURF^n^ implementations are well-documented and freely available for academic and research purposes.

## Methods

Parallel SMote Undersampled Random Forest (parSMURF) is a fast, parallel, and highly scalable algorithm designed to detect deleterious and pathogenic variants in the human genome. The method is able to automatically tune its learning parameters even with large datasets and to nicely scale with big data.

Starting from the presentation of the characteristics and limitations of hyperSMURF [30], in this section we introduce the parallel algorithm parSMURF and its 2 variants named "multi-core parSMURF" (parSMURF^1^) and "multi-node parSMURF" (parSMURF^n^). The first one is suitable for execution on a single workstation that features 1 or more multi-core processors, while the second one is designed for a high-performance computing cluster, where the computation is distributed across several interconnected nodes. Although developed for different hardware architectures, they both share the same core parallelization concepts and the same chain of operations performed on each parallel component of the algorithm. Finally, we discuss the computational algorithms proposed to automatically learn and tune the parSMURF hyper-parameters in order to properly fit the model to the analysed genomic data.

### From hyperSMURF to parSMURF

hyperSMURF is a supervised machine learning algorithm specifically designed to detect deleterious variants where variants associated with diseases are several orders of magnitude less frequent than the neutral genetic variations. hyperSMURF tackles the imbalance of the data using 3 learning strategies:

- balancing of the training data by oversampling the minority class and undersampling the majority class;
- improving data coverage through ensembling techniques;
- enhancing the diversity and accuracy of the base learners through hyper-ensembling techniques.

**Figure 1:**
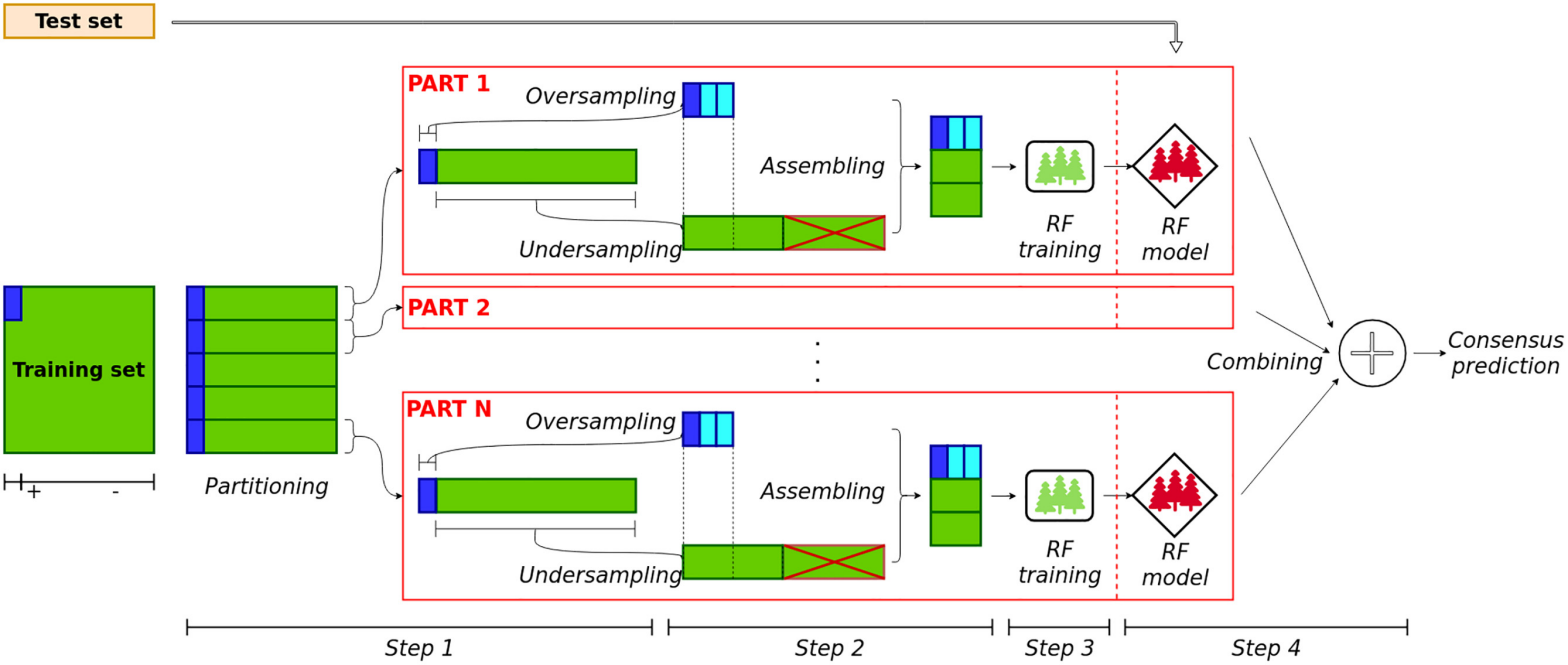
High-level scheme of hyperSMURF. Step 1: partitioning of the training set (the minority/positive class is represented in blue, while the majority/negative class is in green). Step 2: application of oversampling and undersampling approaches, and assembling of the training set. Step 3: training of the random forest models. Step 4: testing and aggregation of prediction outcomes.

The high-level logical steps of the hyperSMURF algorithm are summarized in Fig. 1. At step 1 hyperSMURF creates several sets of training data by using all the available examples of the minority (positive) class and partitioning the set of the majority (negative) class; as a result each set includes all the positive examples and a subset of the majority (negative) class. From this point on, each training set is processed independently. In step 2, examples of the minority class are oversampled through the SMOTE algorithm [32] while examples of the majority class are undersampled according to a uniform distribution. Each training set is now formed by a comparable number of positive and negative examples, and it can be used in step 3 to train the random forest. This process is applied to all the parts of the partition of the original training set, thus generating an ensemble of random forest models. At step 4 all the predictions separately computed by each trained model are finally combined to obtain the “consensus” prediction of the hyper-ensemble.

The behavior of the algorithm strongly depends on the learning hyper-parameters, reported in Table 1, which deeply influence the hyperSMURF performance, as shown in [33]; fine tuning of the learning parameters can dramatically improve prediction performance. Because hyperSMURF is an ensemble of random forests that in turn are ensembles of decision trees, its sequential implementation undergoes a high execution time, especially on large datasets, thus limiting a broad exploration of the hyper-parameter space. Moreover hyperSMURF cannot be easily applied to big data, owing to its time and space complexity issues.

**Table 1:** hyperSMURF learning hyper-parameters

Parameter	Description
nParts	Number of parts of the partition
fp	Multiplicative factor for oversampling the minority class. For instance with fp = 2 two novel examples are synthesized for each positive example of the original dataset, according to the SMOTE algorithm
ratio	Ratio for the undersampling of the majority class. For instance, ratio = 2 sets the number of negative examples as twice the total number of original and oversampled positive examples
k	Number of the nearest neighbours of the SMOTE algorithm
nTrees	Number of trees included in each random forest
mTry	Number of features to be randomly selected at each node of the decision trees included in the random forest

Nevertheless, looking at Fig. 1, we can observe that hyperSMURF is characterized by the following features:

1. the same operations (over- and undersampling, data merging, training, and model generation and prediction evaluation) are performed over different data belonging to different partitions;
2. the operations performed over different data are independent; i.e., there is no interaction between the computation of 2 different partitions;
3. the algorithm does not require any explicit synchronization during the elaboration of 2 or more partitions.

**Figure 2:**
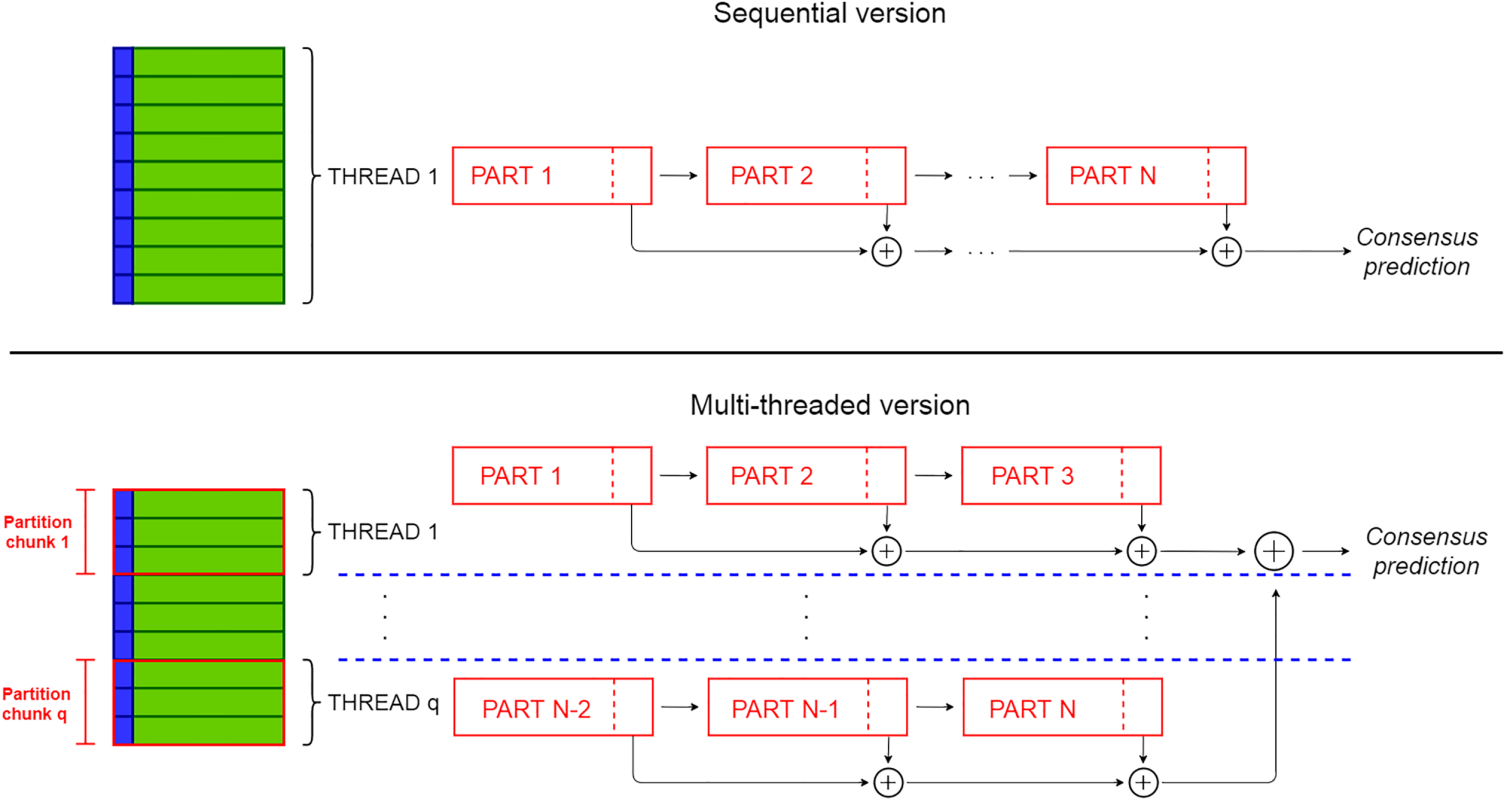
Comparison between the sequential hyperSMURF (top) and multi-core parSMURF^1^ (bottom) execution schemes.

Putting together these observations, we can redesign hyperSMURF, leveraging its intrinsic parallel nature and using state-of-the-art parallel computation techniques. The resulting newly proposed parSMURF algorithm is schematically summarized in Algorithm 1. The parallelization is performed by grouping parts of the partition in "chunks" (see also Fig. 2). The parSMURF parameter *q* (number of chunks) determines at high level the parallelization of the algorithm, i.e., how many chunks can be processed in parallel.

**Algorithm 1:**
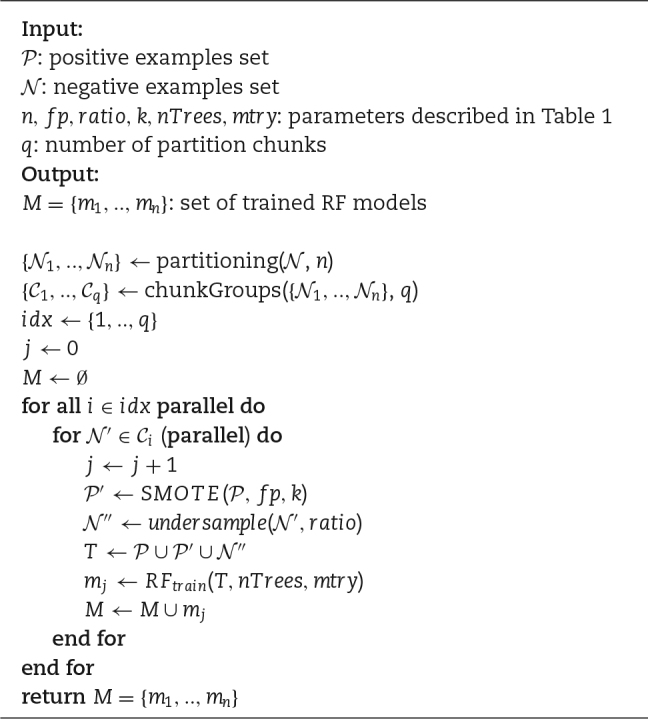
parSMURF algorithm (training)

During training, the main activities of the parSMURF algorithm are executed in parallel for each chunk (outer "for" loop in Algorithm 1). A further level of parallelism can be realized through the inner "for" loop, where each part *N*_i_ of the chunk *C_i_* undergoes a parallel execution. Note however that “parallel” in the inner "for" loop is in brackets to highlight that this second level of parallelization can or cannot be implemented, according to the complexity of the problem and the available underlying computational architecture.

Algorithm 2 also shows that hyper-ensemble predictions conducted during testing can be easily performed through parallel computation: each model can be tested independently over the same test set and the consensus prediction is computed by averaging the ensemble output.

**Algorithm 2:**
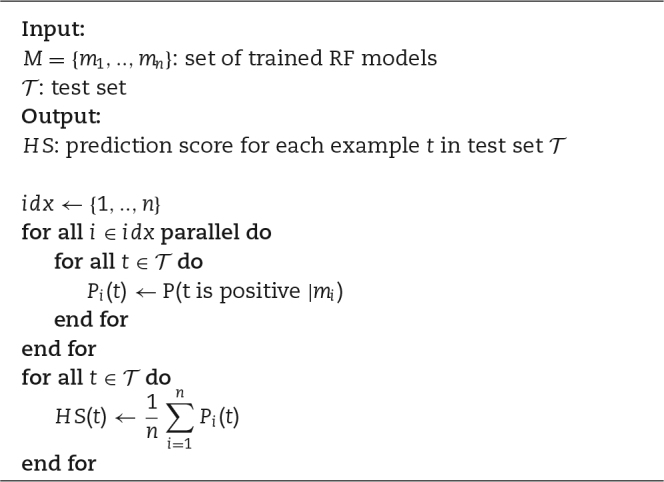
parSMURF algorithm (testing)

### Multi-core parSMURF^1^

The idea on which multi-core parSMURF builds is that all operations performed on the different parts of the partition can be assigned to multiple core/threads and processed in parallel. Namely, given *q* threads, the data parts *N*_1_, …, *N_n_* are equally distributed among threads so that thread *i* receives a subset (chunk) *C_i_* of parts, and processes its assigned parts in sequence. Because each partition chunk is assigned to its own thread, chunk processing is performed in parallel with architectures featuring multiple processing cores.

In Fig. 2 (top) a scheme of the execution of the sequential hyperSMURF is shown: each partition is processed sequentially and the output predictions are accumulated as the computation goes on. On the contrary, with parSMURF^1^ (Fig. 2, bottom), chunks of partitions are assigned to different execution threads and are processed in parallel. To avoid data races, each thread accumulates partial results, and then the master thread collects them once the computation of each thread is ended. Moreover, each thread keeps only a local copy of the subset of the data that is strictly required for its computation; this minimizes memory consumption and, at the same time, does not impose any need for synchronization between concurrent threads.

This scheme is expected to show a remarkable speed-up with the increase of processing cores and the available local memory of the system. Because parallelization occurs at “partition chunk” level, instances of parSMURF^1^ with a reduced partition size benefit only partially from a multi-core execution. On the other hand, partitions characterized by a very high number of parts can theoretically scale well with the number of processing cores, but, unfortunately, current processors have constraints in the number of available cores. Moreover, big data computation may exceed the storage capacity of a single workstation, thus making the application of parSMURF^1^ in this experimental context problematic.

### Multi-node parSMURF^n^

This version of parSMURF has been designed to process big data, to both improve speed-up and make feasible computations that exceed the storage capacity of a single workstation. Moreover parSMURF^n^ allows the fine tuning of the model parameters even when big data are analysed.

#### Architecture

As for the multi-core version, parSMURF^n^ exploits parallelization at partition level, but it also introduces a second level of parallelization: the higher level is performed through the computing nodes of a cluster, i.e., a set of computing machines interconnected by a very fast networking infrastructure; the lower level is realized through multi-threading at single-node level by exploiting the multi-core architecture of each single node of the cluster. In this novel scheme, each node receives a partition chunk, which is processed in parallel with the other chunks assigned to the remaining nodes. Chunks in turn are further partitioned in sub-chunks, distributed among the computing cores available at the local node. Finally an optional third level of parallelization is available by assigning multiple threads to the random forests that process the different parts of the partition (recall that a random forest is in turn an ensemble of decision trees).

The higher level of parallelization leverages the MPI programming paradigm and standard [34] to transfer information among nodes. This programming paradigm requires that several instances of the same program be executed concurrently as different processes (MPI processes) on different nodes interconnected by a network. Being different instances of the same program, each MPI process has its own memory space; therefore intercommunication, i.e., data exchange between processes, occurs explicitly by invoking the corresponding actions, as required by the MPI standard.

parSMURF^n^ adopts a master–slave setting, with a master process coordinating a set of MPI slave processes, also called "worker processes," which in turn manage the partition computation. Master and worker roles are described below:

- the "master process" is responsible for processing the command line parameters, loading data in its memory space, generating partition and chunks, sending the proper subset of data to each worker process (including the test set and the proper fraction of the training set), and finally collecting and averaging their output predictions.
- each "worker process" realizes the computation on the assigned chunk of partitions, generates sub-chunks of its own chunk, and processes them through multi-threading—i.e., distributes the computation of the sub-chunks over the available computing threads—and sends the output predictions back to the master process.

**Figure 3:**
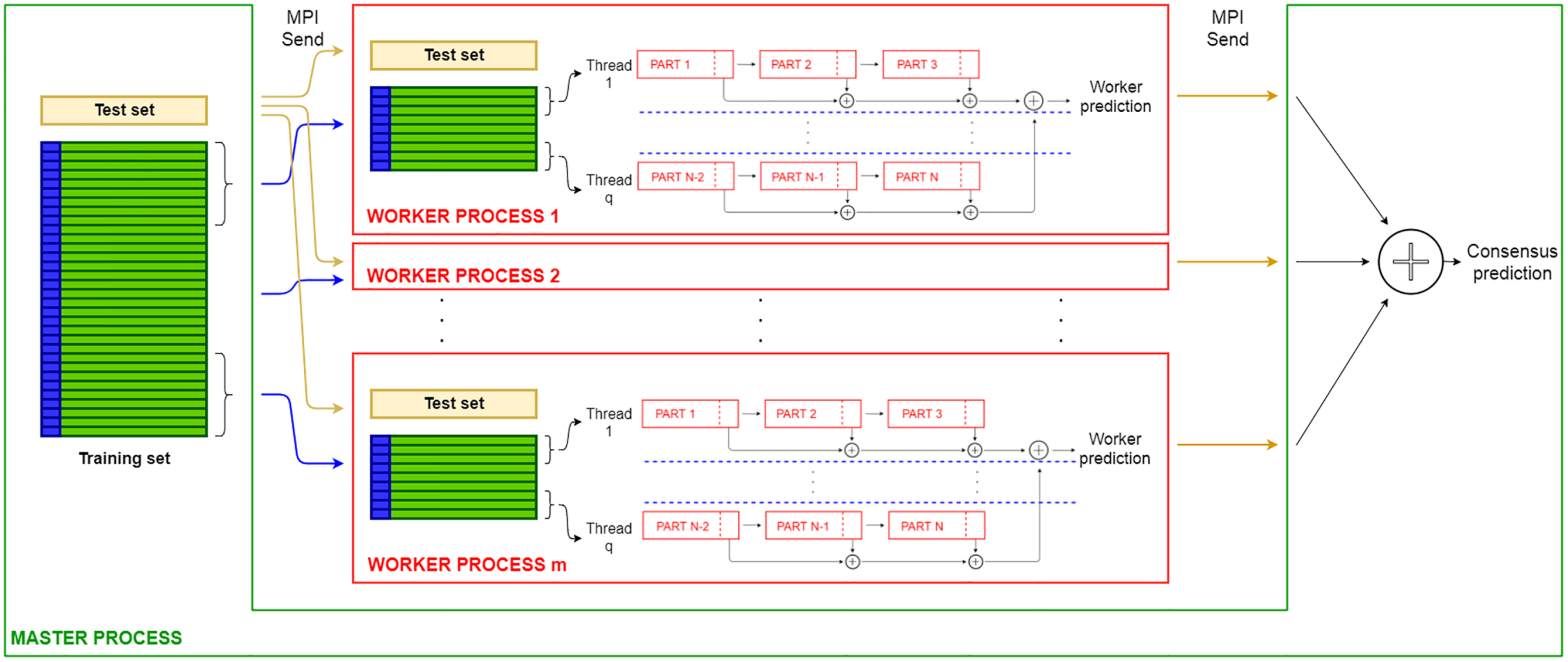
High-level scheme of the multi-node parSMURF^n^ implementation.

We point out that in principle parSMURF^n^ can also be executed on a single machine, where multiple copies of the same program are processed by the available cores, but in this case it has the same limitations as parSMURF^1^. Fig. 3 provides a high-level scheme of the execution of parSMURF^n^.

#### *parSMURF* ^*n*^ intercommunication

**Figure 4:**
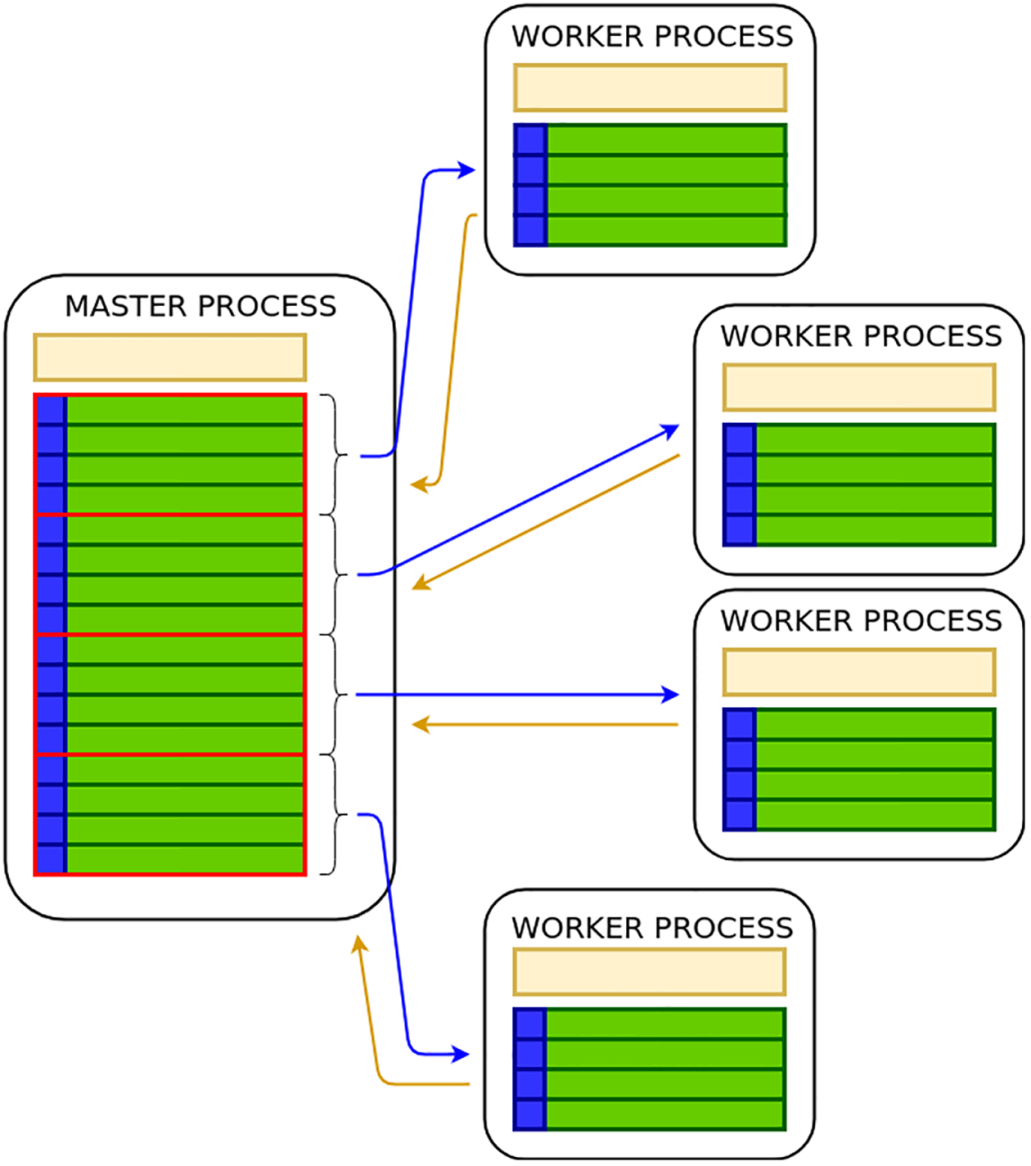
High-level intercommunication scheme between MPI processes in multi-node parSMURF^n^. Blue arrows represent data flows from the master process to worker processes (different chunks of partitions and the same test set) and yellow arrows represent data flows from the worker processes to the master (output predictions).

Fig. 4 shows a schematic view of the intercommunication between parSMURF MPI processes.

The computation in the worker processes is performed as in the multi-core version of parSMURF, except for a slight difference in the subsampling of the majority class, because this operation is no longer executed by the worker processes but by the master instead. Indeed, by observing that subsampling requires some examples to be discarded, there is no need of sending to the worker processes an entire part of the partition but only the selected subset of examples. This design choice minimizes the amount of data to be sent to a worker process because for each partition only the positive samples (that are going to be oversampled in the worker process) and the already undersampled negative examples are sent to the worker processes.

In an ideal parallel application, computing nodes should never interact because every data exchange creates latencies that affect the overall "occupancy"—i.e., the ratio between the amount of time a computing node is processing data and the total execution time. However, in real applications this rarely happens, and data have to be exchanged between processes. As a general rule, communication should be minimal in number and maximal in size because the following equality holds:

$$\begin{equation*}
t_{\mathrm{tot}} = t_{\mathrm{start}} + d \times t_{\mathrm{trn}},
\end{equation*}$$

where *t*_tot_is the total time for the data send, *d* is the amount of data in bytes to be transferred, *t*_trn_ is the time required to transfer 1 B of data, and *t*_start_ is the time required by the interconnecting networking system for beginning a communication between nodes. *t*_start_ is constant; therefore transferring data as a big chunk is generally faster than for several smaller ones because the *t*_start_ penalty is paid only once in the former case. However, in real-world MPI parallel applications, the main objective is to parallelize computation to speed up execution, and maximum efficiency is achieved by overlapping data transmission and computation. This is easier to obtain when data are "streamed," i.e., sent in small chunks that are consumed as soon as they reach the receiver MPI process; in this way we can minimize the inactivity time of a node, waiting for data to be received.

#### Maximizing *parSMURF*^*n*^ performance

For maximizing performance, parSMURF^n^ adopts the following strategies to find the optimal balance between the size and number of data transmissions:

- maximize occupancy,
- reduce the amount of data sent or broadcast,
- minimize latency.

To maximize occupancy, the master process does not send the entire chunk of partitions to each worker process in a big data send; instead, parts are sent one by one. This choice is ideal in the context of multi-threading in worker processes: supposedly, given a partition with *n* parts and a number *x* of computing threads assigned to a working process, the master at first sends to each worker process *x* parts of its assigned chunk. When a worker thread finishes the computation of a part, another one is sent by the master for processing. This process goes on until the chunk is exhausted.

To reduce the amount of data sent or broadcast—i.e., 1 MPI process sending the same data to all the other processes—for each part, the master process assembles an array having all the relevant data required for the computation, i.e., the positive and negative examples (already subsampled, as stated earlier) and the parameters needed for the computation. Hence with just 1 MPI send operation, a part of the partition with its parameters is transferred to the worker process. Also, partial results of the predictions are locally accumulated inside each worker and sent to the master once the jobs for the assigned chunk are finished.

To minimize latency, the assembly of the data to be sent is multi-threaded in the master process. In instances characterized by relatively small datasets and a high number of parts in the partition, it may happen that the master could not prepare and send data fast enough to keep all the worker processes busy. For instance, a thread in a worker process may finish the computation for a part before data for the next one arrive, leaving the thread or the entire process inactive. This has been solved by spawning a number of threads in the master process equal to the number of worker processes the user has requested, each one assigned for preparing and sending the data to the corresponding worker. However, because memory usage in the master process can be greatly affected, an option is provided for disabling multi-threading in the master. In this case, only 1 thread takes care of this task and parts are sent in round-robin fashion to the working processes; this has been experimentally proven to be effective for those instances that require a particularly high memory usage.

### Hyper-parameter tuning

As in most machine learning methods, the accuracy of the predictions of the parSMURF models is directly related to the set *S* of hyper-parameters that control its learning behaviour. Hence, to maximize the usefulness of the learning approach, the value of each hyper-parameter of the set *S* must be chosen appropriately. In parSMURF the hyper-parameter set is composed of the 6-tuple of parameters reported in Table 1. Each parameter is discretized and constrained between a maximum and minimum value; hence the hyper-parameter space ${\mathcal {H}}$ is a discrete 6-dimensional hypercube. The validation procedure for the evaluation of each $h \in {\mathcal {H}}$ is the internal cross-validation (CV) process, and the objective function (performance metrics) that has to be maximized is the area under the precision recall curve (AUPRC).

parSMURF features 2 modes for automatically finding the combination of hyper-parameters $h_0 \in \mathcal {H}$ that maximizes the model accuracy (parameter auto-tuning). The first strategy is a grid search over *H*, where each $h \in \mathcal {H}$ is evaluated by internal CV. This strategy is generally capable of finding values close to the best combination of hyper-parameters, but it is very computationally intensive and is hindered by the curse of dimensionality. The second strategy is based on a BO, which iteratively builds a probabilistic model of the objective function $f: {\mathcal {H}} \rightarrow \mathbb {R}^+$ (in parSMURF, the AUPRC) by evaluating a promising hyper-parameter combination at each iteration and stopping when a global maximum of *f* is obtained. This procedure is less computationally intensive than the grid search and may also outperform the latter in terms of prediction effectiveness. The BO is based on [35] and its implementation “Spearmint-lite” [36] is included in the parSMURF package.

The whole procedure is summarized in Algorithm 3. Briefly, parSMURF provides the automatic tuning of the hyper-parameters in a context of internal *n*-fold CV, and the BO is invoked in the while loop. At each iteration, a new hyper-parameter combination $h \in \mathcal {H}$ is generated by taking into account all the previously evaluated *h*. A new model is then trained and tested in the internal CV procedure by using the newly generated *h*. The quality of the prediction is evaluated by means of AUPRC, and the tuple (*h*, eval) is submitted back to the BO for the generation of the next *h*. The while loop ends when the BO finds a probable global maximum (no further improvement in the error evaluation) or when the maximum number of iterations is reached. Grid search works in a similar way, but all $h \in \mathcal {H}$ are exhaustively tested in the internal CV phase.

**Algorithm 3:**
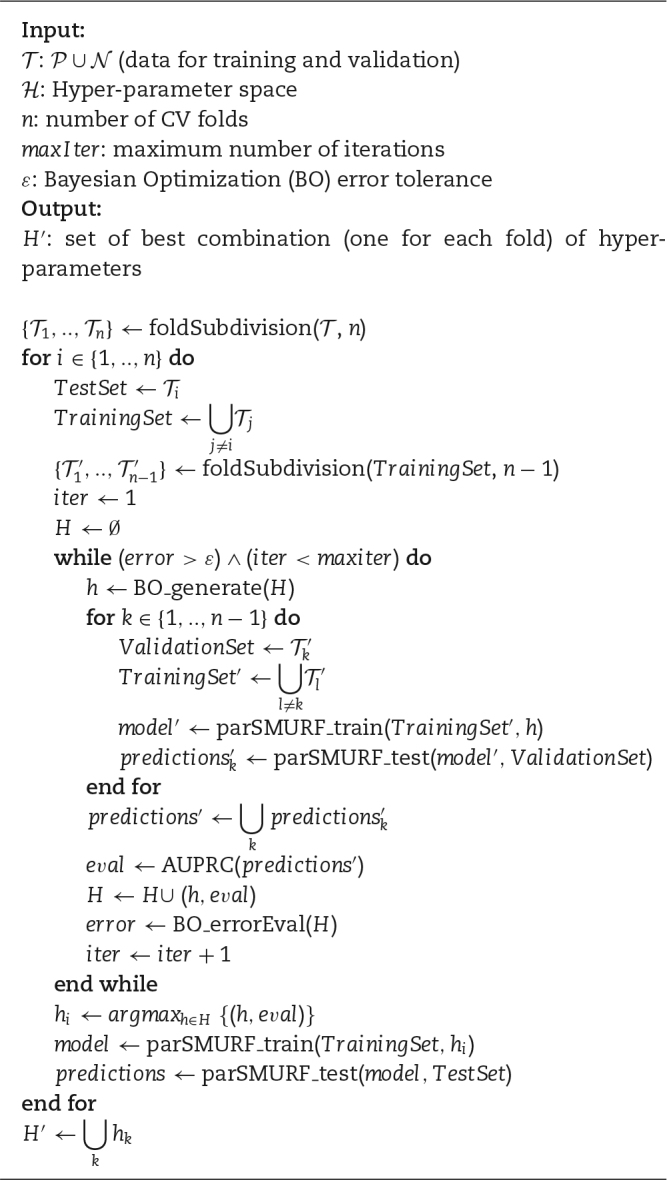
Automatic hyper-parameters tuning in parSMURF featuring Bayesian Optimization.

## Results and Discussion

We applied parSMURF to both synthetic and real genomic data to show that the proposed method is able to:

- scale nicely with big data;
- auto-tune its learning parameters to optimally fit the prediction problem under study;
- improve on hyperSMURF as well as other state-of-the-art methods in the prediction of pathogenic variants in Mendelian diseases and of regulatory GWAS hits in the GWAS Catalog.

All the experiments have been performed on the Cineca Marconi Supercomputing system [37], specifically using its Lenovo NeXtScale architechture, with 720 nodes, each one having 128 GB of RAM and 2 × 18-cores Intel Xeon E5-2697 v4 (Broadwell) CPUs at 2.30 GHz. The interconnecting infrastructure is an Intel Omnipath featuring 100 GB/s of networking speed and a fat-tree 2:1 topology.

### Datasets

Genomic data are highly imbalanced toward the majority class because the single-nucleotide variants (SNVs) annotated as pathogenic represent a tiny minority of the overall genetic variation. Synthetic data have also been generated to obtain a high imbalance between positive and negative examples, in order to simulate the imbalance that characterizes several types of genomic data.

Synthetic data have been randomly generated using a spherical Gaussian distribution for each of the 30 features. Among them only 4 are informative in the sense that the means of positive and negative examples are different, while all the other features share the same mean and standard deviation with both positive and negative examples. Synthetic data, as well as the R code for their generation, are available from the GitHub repository [38].

As an example of application of parSMURF to real genomic data, we used the dataset constructed in [29] to detect SNVs in regulatory non-coding regions of the human genome associated with Mendelian diseases. The 406 positive examples are manually curated and include mutations located in genomic regulatory regions such as promoters, enhancers, and 5′ and 3′ untranslated regions (UTRs). Neutral (negative) examples include >14 millions of SNVs in the non-coding regions of the reference human genome differing, according to high-confidence alignment regions, from the ancestral primate genome sequence inferred on the basis of the Ensembl Enredo-Pechan-Ortheus whole-genome alignments of 6 primate species [39], and not including variants present in the most recent 1000 Genomes data [6] with frequency >5% to remove variants that had not been exposed for a sufficiently long time to natural selection. The imbalance between positive (mutations responsible for a Mendelian disease) and negative SNVs amounts to ∼1:36,000. The 26 features associated with each SNV are genomic attributes ranging from G/C content and population-based features to conservation scores, transcription, and regulation annotations (for more details, see [29]).

We finally analysed genome-wide association studies (GWAS) data to detect 2,115 regulatory GWAS hits downloaded from the National Human Genome Research Institute (NHGRI) GWAS catalog [40], and a set of negatives obtained by randomly sampling 1/10 of the negative examples of the Mendelian dataset, according to the same experimental set-up described in [30], thus resulting in an imbalance between negative and positive examples of ∼1:700. We predicted chromatin effect features directly from the DNA sequence using DeepSEA convolutional networks [18]; in this way we obtained 1,842 features for each SNV, as described in [30], and we used those features to train parSMURF.

Table 2 summarizes the main characteristics of both the synthetic and genomic data used in our experiments.

**Table 2:** Summary of the datasets used in the experiments

Name	No. of samples	No. of features	No. of positive samples	Imbalance ratio
synth_1	10^6^	30	400	1:2,500
synth_2	10^7^	30	400	1:25,000
synth_3	5 × 10^7^	30	1,000	1:50,000
Mendelian	14,755,605	26	406	1:36,300
GWAS	1,477,630	1,842	2,115	1:700

Datasets are highly imbalanced towards the negative class.

### *parSMURF* scales nicely with synthetic and genomic data

Experiments reported in this section follow the classic experimental set-up for the evaluation of the performance of parallel algorithms [41]. In particular, because our executions use multiple CPUs concurrently, we use speed-up and efficiency to analyse the algorithm performance by measuring both the sequential and parallel execution times.

By denoting with *T_s_* the run-time of the sequential algorithm and with *T*_p_ the run-time of the parallel algorithm executed on *P* processors, the speed-up and efficiency are defined, respectively, as

$$\begin{equation*}
S=\frac{T_s}{T_p}\qquad \text{and}\qquad E=\frac{T_s}{T_p \times P}.
\end{equation*}$$

#### Speed-up and efficiency analysis with synthetic data

##### Experimental set-up

For every synthetic dataset, we run parSMURF^1^ and parSMURF^n^ several times by varying the number of threads (for both the multi-core and multi-node versions) and the number of MPI worker processes assigned to the task (for the multi-node version only). More precisely the number of threads n.thr varied in n.thr ∈ {1, 2, 4} for synth_1 and synth_2 datasets, while for synth_3 n.thr ∈ {1, 2, 4, 8, 16, 20}. Moreover we considered a number of MPI processes n.proc in the range n.proc ∈ {1, 2, 4, 8} for synth_1 and synth_2, and n.proc ∈ {1, 2, 4, 6, 8, 10} for synth_3.

For each run we collected the execution time and evaluated the speed-up and efficiency: the *T*_s_ sequential time of formulas (1) and (2) has been obtained by running parSMURF^1^ with 1 thread, hence obtaining a pure sequential run.

All experiments were executed using a 10-fold CV setting. The learning hyper-parameters used in each experiment are the following:

- synth_1: nParts = 128, fp = 1, ratio = 1, *k* = 5, nTrees = 128, mTry = 5;
- synth_2: nParts = 64, fp = 1, ratio = 1, *k* = 5, nTrees = 32, mTry = 5;
- synth_3: nParts = 128, fp = 1, ratio = 5, *k* = 5, nTrees = 10, mTry = 5

For each synthetic dataset we run experiments considering all the different numbers of threads n.thr for parSMURF^1^, while for parSMURF^n^ we run different hyper-ensembles considering all the possible combinations of n.thr and n.proc.

##### Results and discussion

**Figure 5:**
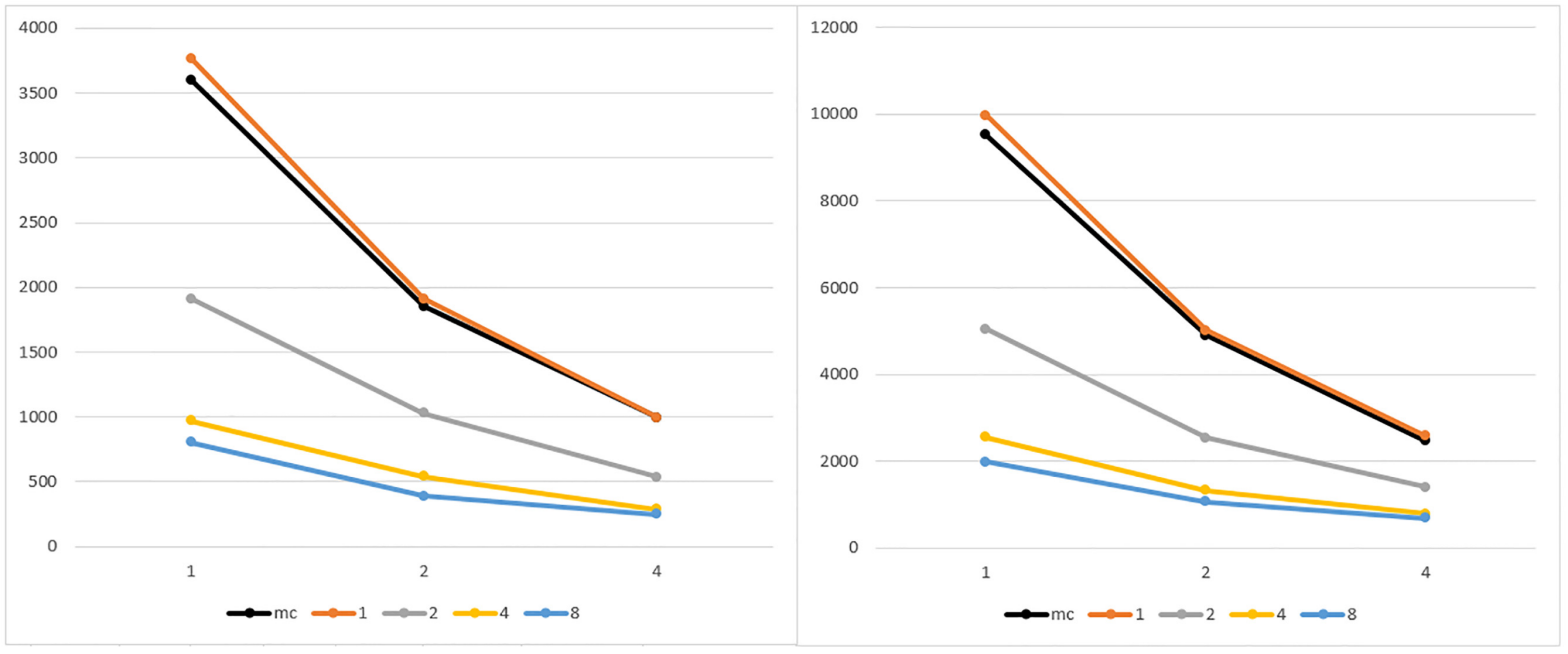
Execution time of parSMURF^1^ and parSMURF^n^ on the synthetic datasets synth_1 (left) and synth_2 (right). The x-axis shows the number of threads for each MPI process; the y-axis, execution time in seconds; experiments are grouped by the number of MPI processes. The black line represents the multi-thread version, while orange, grey, yellow, and light blue show the MPI version with 1, 2, 4, and 8 MPI processes, respectively.

Fig. 5 reports the results for the batch of executions with the synth_1 and synth_2 datasets. Results are grouped by the number of MPI working processes (each line represents 3 runs obtained by keeping the number of MPI processes fixed and by varying the number of threads per process).

Both graphs show that the multi-core and the multi-node implementation of parSMURF introduce a substantial speed-up with respect to the sequential version (the point in the black line with 1 thread in the abscissa). Note that in Fig. 5 the black line represents parSMURF^1^, and the orange line, parSMURF^n^; their execution time is very similar because both use the same overall number of threads, with a small overhead for the MPI version due to the time needed to set up the MPI environment. Table 3 shows that the speed-up achieved by parSMURF^n^ is quasi-linear with respect to the overall number of aggregated threads (i.e., the product n.thr × n.proc) involved in the computation, at least up to 16 threads. By enlarging the number of aggregated threads we have a larger speed-up, but a lower efficiency, due to the lower number of parts of the partition assigned to each thread and to the larger time consumed by the MPI data intercommunication.

**Table 3:** Execution time and speed-up of parSMURF^n^ with synth_1 and synth_2 datasets.

Aggregated threads	synth_1 time (s)	synth_1 speed-up	synth_2 time (s)	synth_2 speed-up
1	3,768.59		9,981.82	
2	1,910.19	1.97	5,020.18	1.99
4	571.56	3.88	2,539.10	3.93
8	542.35	6.95	1,329.74	7.51
16	288.82	13.05	788.31	12.66
32	248.84	15.18	686.41	14.54

Threads are counted as “aggregated” in the sense that they are the product of the number of MPI processes with the number of threads for each process. All executions have been performed with a 10-fold cross-validation setting.

**Figure 6:**
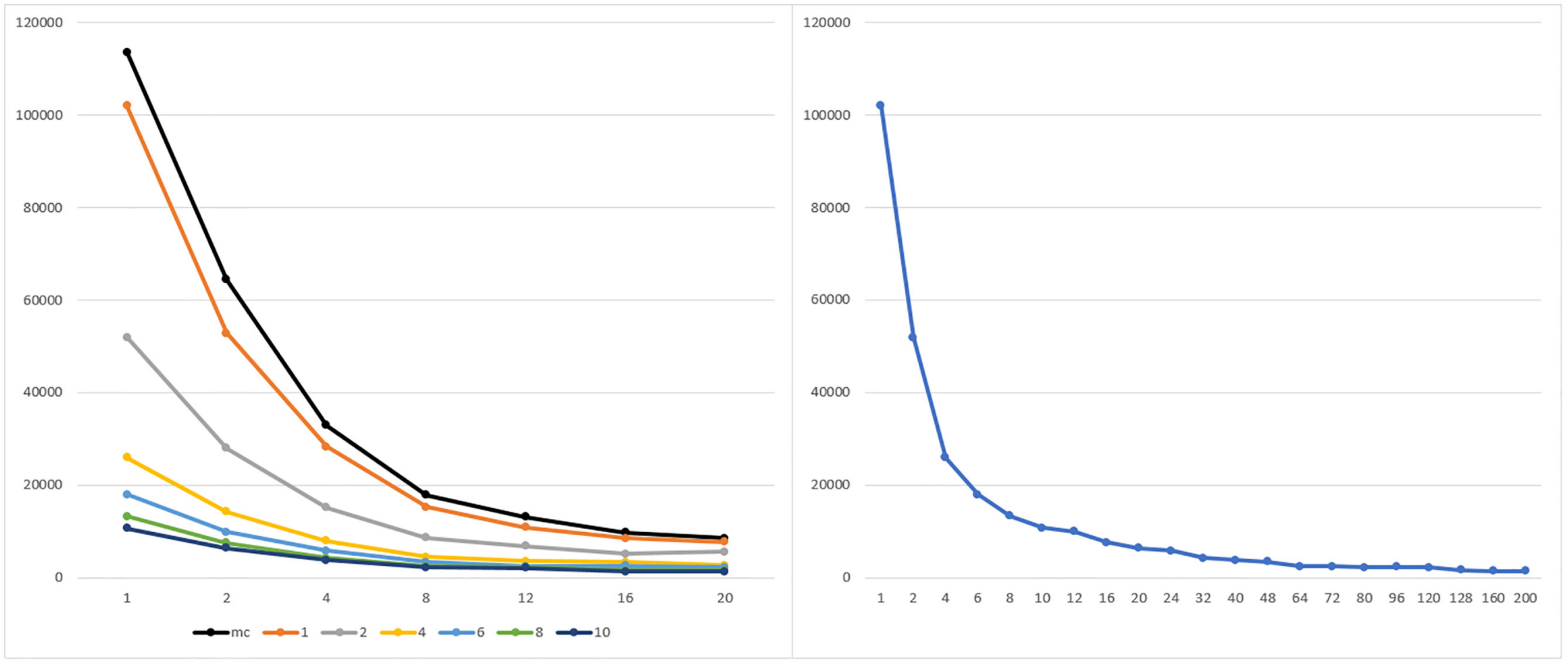
Execution time of parSMURF^1^ and parSMURF^n^ on the synthetic dataset synth_3. Left: The x-axis shows the number of threads for each MPI process; the y-axis, execution time in seconds. Experiments are grouped by the number of MPI processes. The black line represents the multi-thread version, while orange, grey, yellow, light blue, green, and blue show the MPI version with 1, 2, 4, 6, 8, and 10 MPI processes, respectively. Right: Results are grouped by total number of threads (n.thr × n.proc). When a combination is obtainable in >1 way only the best time is considered.

Results with the synthetic dataset synth_3, which includes 50 million examples, confirm that parSMURF scales nicely also when the size of the data is significantly enlarged. Indeed Fig. 6 (left) shows that by increasing the number of processes and threads we can obtain a considerable reduction of the execution time. These results are confirmed by grouping the execution time with respect to the aggregated number of threads, i.e., the product n.thr × n.proc (Fig. 6 right).

**Figure 7:**
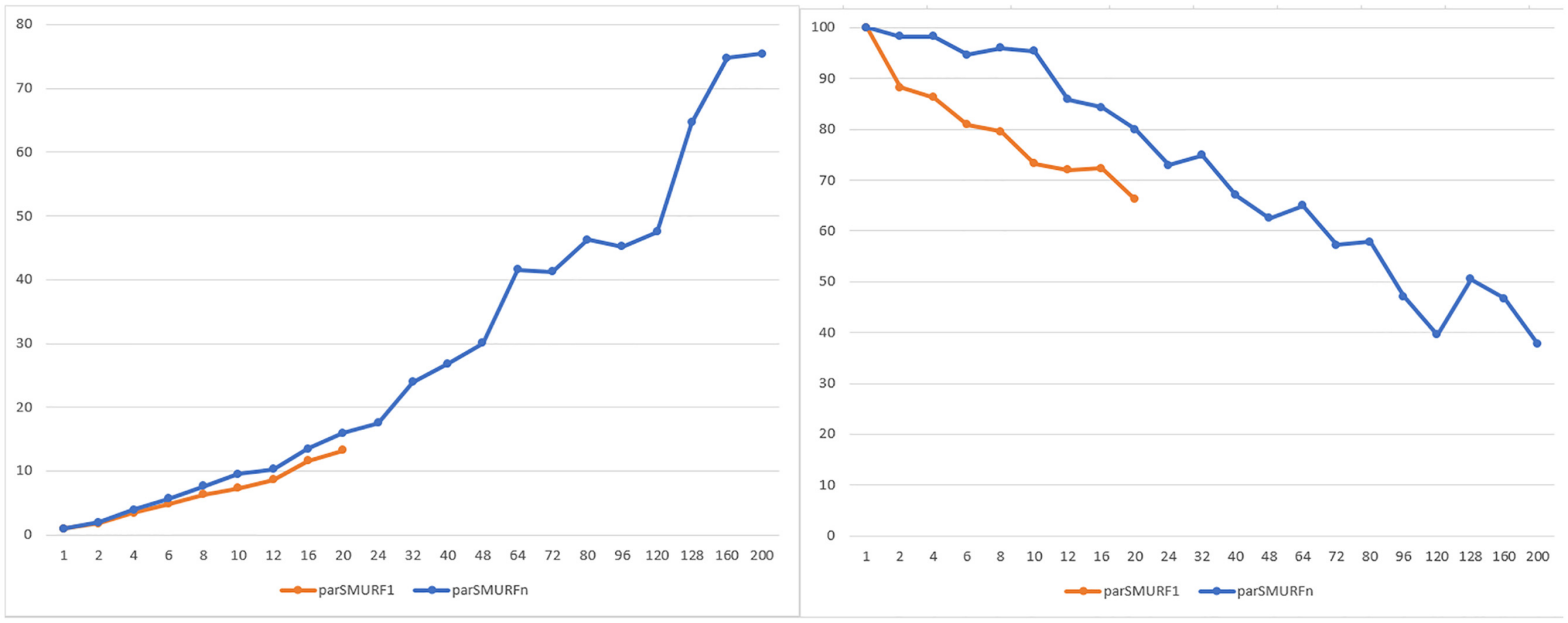
Left: Speed-up of parSMURF^1^ and parSMURF^n^ on the synthetic dataset synth_3. The x-axis shows the “aggregated” number of threads; the y-axis, speed-up. Right: Efficiency of parSMURF with the synthetic dataset synth_3. The x-axis shows the aggregated number of threads; the y-axis, efficiency in percentage.

Fig. 7 shows the speed-up (left) and efficiency (right) obtained with this dataset; results are again grouped by the aggregated number of threads. Note that with this large dataset we can obtain a larger speed-up, even if, as expected, it is at the expense of the overall efficiency.

Different research works showed contradictory results for the comparison of the performance of pure multiprocess MPI, pure multi-thread OpenMP, and hybrid MPI-OpenMP implementations of the same algorithm, showing that several factors, such as algorithms, data structures, data size, hardware resources, and MPI and OpenMP library implementations, influence their performance [42–49].

**Figure 8:**
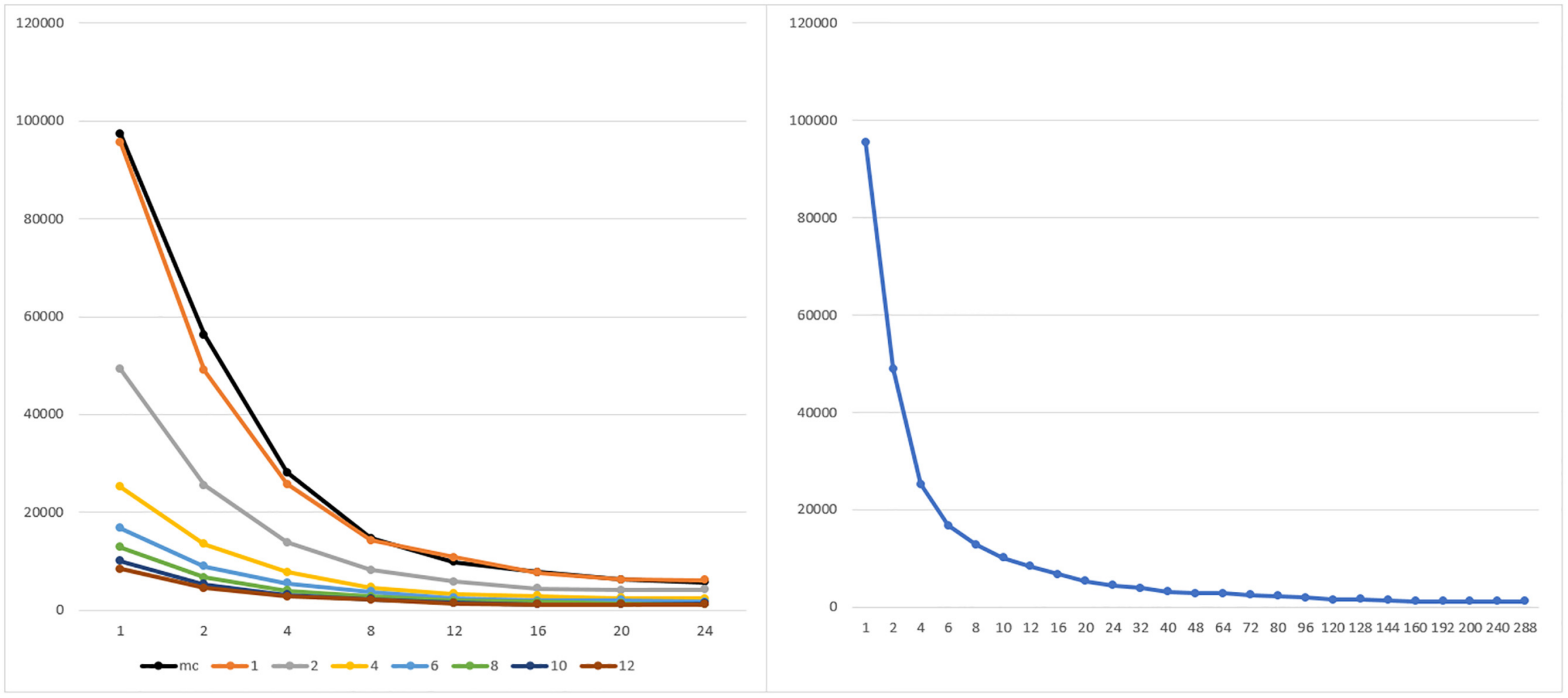
Execution time of parSMURF^1^ and parSMURF^n^ on the Mendelian dataset. Left: The x-axis shows the number of threads for each MPI process; the y-axis, execution time in seconds. Experiments are grouped by number of MPI processes. The black line represents the multi-thread version, while orange, grey, yellow, light blue, green, blue, and brown show the MPI version with 1, 2, 4, 6, 8, 10, and 12 MPI processes, respectively. Right: Results are grouped by total number of threads (n.thr × n.proc).

**Figure 9:**
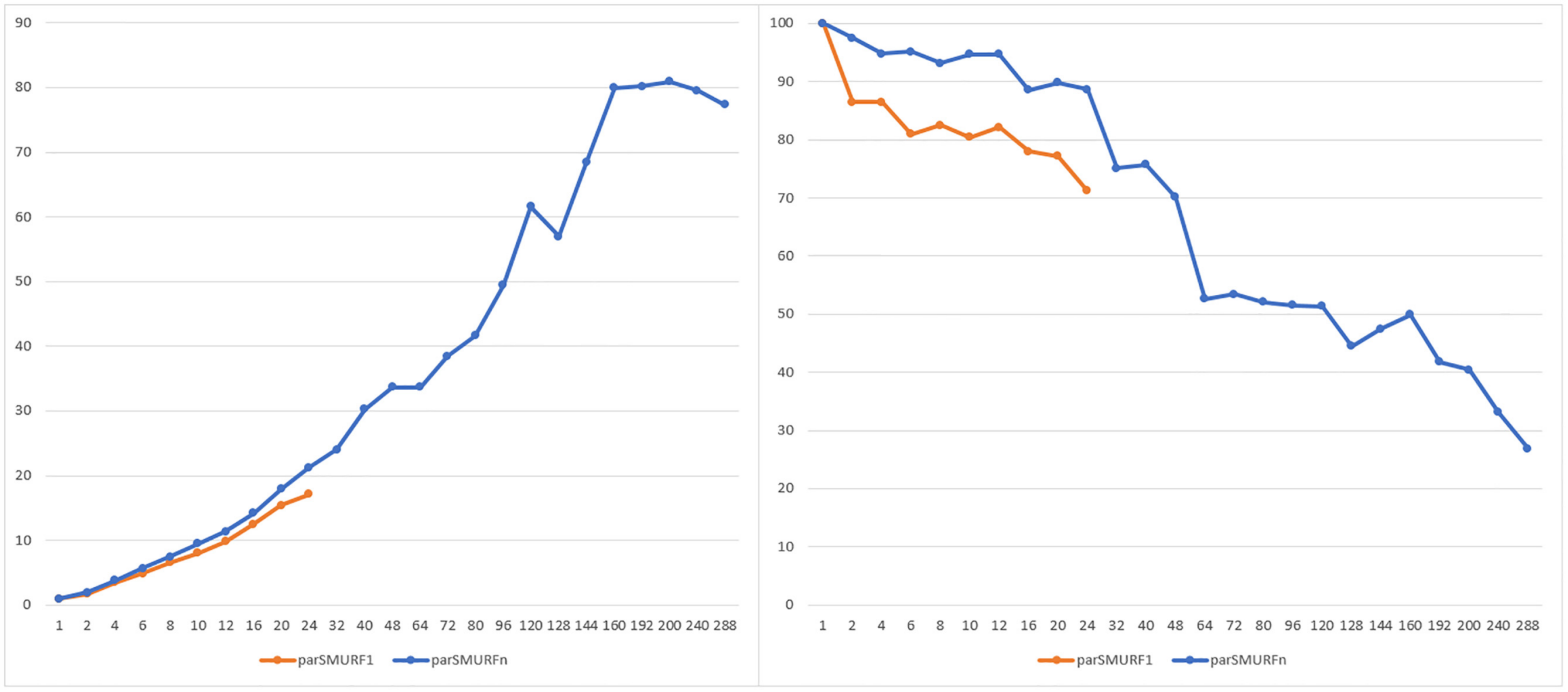
Left: Speed-up of of parSMURF^1^ and parSMURF^n^ with the Mendelian dataset. The x-axis shows the “aggregated” number of threads; the y-axis, speed-up. Right: Efficiency of parSMURF with the Mendelian dataset.The x-axis shows the aggregated number of threads; the y-axis, efficiency in percentage.

Regarding our experiments, from Figs 6 and 8, we can notice how, in some cases, a pure MPI implementation may outperform a heterogeneous MPI-multi-thread or a pure OpenMP-multi-thread implementation. However, more in general, parSMURF^n^ allows a higher degree of parallelism, thus resulting in a larger speed-up and efficiency with respect to the pure multi-thread parSMURF^1^ (Figs 7 and 9).

#### Speed-up and efficiency analysis with genomic data

To show how parSMURF performs in term of speed-up and efficiency on a real genomic dataset, we carried out the same batch of experiments as in the previous section using this time the Mendelian dataset.

Fig. 8 shows the execution time of parSMURF^1^ and parSMURF^n^ as a function of the “aggregated number of threads,” i.e., the product of the number of MPI processes and the number of threads per process. As expected, the results show a substantial decrement in execution time with respect to the number of the aggregated threads.

Fig. 9 shows the speed-up and efficiency of parSMURF: on the x-axis of both graphs, threads are counted as “aggregated”; i.e., the total number of threads is computed by multiplying the number of processes by the number of threads assigned to each process. For the evaluation of speed-up and efficiency, parSMURF^1^ with only 1 computing thread has been used as reference for obtaining the computation time of the sequential version.

The maximum speed-up of parSMURF^1^ is ∼17 times, with the execution time decreasing from 97,287 seconds for the sequential version to 5,695 seconds for the multi-threaded version using 24 cores. The speed-up of parSMURF^n^ is even better, with a maximum speed-up of 80 times (1,181 seconds execution time) obtained using 10 MPI processes with 20 computing threads each. The graph shows that both parSMURF^1^ and parSMURF^n^ follow the same trend in the increment of the speed-up, but the multi-thread version is limited to 24 threads (each assigned to a different core), while parSMURF^n^ continues this trend up to 288 threads, reaching a speed-up saturation level of 80 times. As just observed with synthetic data (Fig. 7), the efficiency tends to decrease with the number of aggregated threads.

To summarize, both experiments with synthetic and genomic data show that parSMURF scales nicely with large data and achieves a considerable speed-up that allows its application to big data analysis and to the fine tuning of learning parameters.

### Auto-tuning of learning parameters improves prediction of pathogenic non-coding variants

The speed-up introduced by parSMURF allows the automatic fine tuning of its learning parameters to improve predictions on real genomic data. Indeed, as preliminarily shown in [33], fine tuning of hyperSMURF learning parameters can boost the performance on real data.

To this end we run parSMURF^n^ on the Cineca Marconi cluster using auto-tuning strategies to find the best learning parameters for both the prediction of pathogenic non-coding SNVs in Mendelian diseases and for the prediction of GWAS hits that overlap with a known regulatory element.

We compared the auto-tuned results only with those obtained with the default learning parameters of hyperSMURF, because our previous studies showed that hyperSMURF outperformed other methods, such as CADD [14], GWAVA [27], Eigen [19], and DeepSea [18] with both Mendelian diseases and GWAS hits data [30], and, above all, because we are more interested in showing a proof-of-concept of the fact that auto-tuning of learning parameters may lead to better performance in a real genomic problem.

#### Experimental set-up

Generalization performance has been evaluated through an external 10-fold “cytogenetic band-aware” CV setting. This CV technique ensures that variants occurring nearby in a chromosome (i.e., in the same cytogenetic band) do not occur jointly in the training and test sets and thereby bias results, because nearby variants may share similar genomic features [30]. Learning parameters were selected through a grid search realized through a 9-fold internal CV; i.e., for each of the 10 training sets of the external CV, their 9 cytogenetic band-aware folds have been used to select the best learning parameters and to avoid putting contiguous variants located in the same cytoband both in training and in the validation set.

This experimental set-up is computationally demanding, but by exploiting the different levels of parallelism available for parSMURF^n^ we can obtain a sufficient speed-up to experiment with different hyper-ensembles having different sets of learning parameters.

Performance of the prediction is evaluated via the area under the receiver operator characteristic curve (AUROC) and the area under the precision-recall curve (AUPRC). Because data are highly unbalanced, we outline that it is well-known that in this context AUPRC provides more informative results [50,51].

#### Improving predictions of pathogenic Mendelian variants

We at first executed hyperSMURF with default parameters (specifically, nParts = 100, fp = 2, ratio = 3, *k* = 5, nTrees = 10, and mTry = 5) in a context of 10-fold cytogentic-band aware CV because this experiment is used as reference for the next steps.

We tested the auto-tuning feature by performing a grid search over the hyper-parameter space $\mathcal {H}_g$ defined in Table 4, col. 2. Such hyperspace provides 576 possible hyper-parameter combinations $h \in \mathcal {H}_g$. Then, we applied the auto-tuning strategy based on the BO, by defining the hyper-parameter space $\mathcal {H}_b$ as in Table 4, col. 3.

**Table 4:** Hyper-parameter spaces for grid search ($\mathcal {H}_g$) and Bayesian optimizer ($\mathcal {H}_b$) used for the auto-tuning on the Mendelian dataset

Parameter	$\mathcal {H}_g$	$\mathcal {H}_b$
nParts	{10, 50, 100, 300}	[10, 300]
fp	{1, 2, 5, 10}	[1, 10]
ratio	{1, 2, 5, 10}	[1, 10]
k	{5}	{5}
nTrees	{10, 20, 100}	[10, 100]
mTry	{2, 5, 10}	[2, 10]

To fully exploit the scalability of parSMURF, we launched the grid search with the following configuration: 10 instances of parSMURF^n^, one for each fold of the external CV, each one having 10 worker processes, with 6 dedicated threads for processing the different parts of the partition plus a further 4 threads for each random forest training and testing. Hence, for the grid search, we used a total of 2,400 CPU cores. Because the Bayesian auto-tuning procedure is less computationally intensive, we chose a more conservative approach on resource utilization for this experimental set-up: we launched 1 instance of parSMURF^n^ having 24 worker processes with 16 threads for the partitions and 1 for the random forest training and testing. Folds of the external CV are processed sequentially. Therefore, for the Bayesian optimization set-up we used 384 CPU cores.

At the end of this phase, for each optimization strategy, parSMURF returns the best hyper-parameter combination for each fold. We then executed 10 repetitions of the external CV using the default parameters, 10 using the best hyper-parameters found by the grid search, and 10 using the best hyper-parameters found by the BO. Performance in terms of AUROC and AUPRC is measured for each repetition and then averaged.

Performance improvements relative to the above parameter-tuning experiments and their execution times are summarized in Table 7. Results in cols 4 and 5 show a significant improvement in the prediction performance in terms of AUPRC using both optimization strategies (Wilcoxon rank sum test, α = 10^−6^). On the other hand, AUROC is very high in all the experiments, confirming that this metric is not sufficient for the evaluation of prediction performance in the context of highly unbalanced datasets. Supplementary Figs S1 and S2 show the computer ROC and precision-recall curves of both hyperSMURF and parSMURF. Also, the BO proves to be effective in both improving the prediction performance and reducing the computational time: although slightly lower, predictions are comparable to the grid search, but they are obtained at a fraction of the computational power required by the latter. As a matter of fact, the CPU time required by the entire grid search counted >120,000 hours, compared with 16,000 hours used by the Bayesian optimization strategy.

Table 7 reports mean AUROC and AUPRC measured on the training sets: results show that the ratio between training and test AUROC or AUPRC is quite similar between hyperSMURF and parSMURF, and even if, as expected, results on the training sets are better, they are comparable to those obtained on the test data. These results show that performance improvement is not due to overfitting but to a proper choice of the hyper-parameters well-fitted to the characteristics of the problem.

To assess whether the difference in performance between hyperSMURF and parSMURF can be related to their different capacity of selecting the most informative features, we measured the Spearman correlation between both hyperSMURF and parSMURF scores with each of the 26 features used to train the hyper-ensembles for all the examples of the dataset. Table S3 in the Supplementary Information reports the correlation between the true labels of the examples and the predictions obtained by hyperSMURF using the default set of hyper-parameters, and parSMURF with the default, grid-optimized, and Bayesian-optimized set of hyper-parameters. We found that hyperSMURF and parSMURF achieved very similar Spearman correlation on each feature (the Pearson correlation between the vectors of Spearman correlations of hyperSMURF and parSMURF is ∼0.98). Both hyperSMURF and parSMURF obtained the largest Spearman correlation coefficients for features related to the evolutionary conservation of the site (e.g., vertebrate, mammalian, and primate PhyloP scores) and for some epigenomic features (histone acetylation, methylation, and DNAse hypersensitivity). Again, these results show that it is unlikely that the improved performance of parSMURF can be explained through its better capacity to select the most informative features, but instead by its capacity of auto-tuning its learning hyper-parameters and its capacity to find a model that better fits the data.

In addition, in Table 6 some examples of pathogenic variants that have been ranked remarkably better by parSMURF than hyperSMURF are reported. Further details about these variants are presented in Table S6 of the Supplementary Information.

#### Prediction performance of *parSMURF* with an independent Mendelian test set

We collected novel Mendelian pathogenic variants by adding 64 newly positive (pathogenic) non-coding variants manually annotated according to a comprehensive literature review. We included only those variations and publications judged to provide plausible evidence of pathogenicity (Supplementary Table 7). As negative examples we used common variants downloaded from NCBI [52], i.e., variants of germline origin and having a minor-allele frequency ≥0.01 in ≥1 major population, with ≥2 unrelated individuals having the minor allele, where major populations are those included in the 1000 Genomes Project [53]. We selected only those variants that lie in non-coding regions using Jannovar [54]. The final number of negatives (∼3 million examples) has been randomly sampled in such a way that the ratio positives/negative in the original and in the new Mendelian dataset used for validation is approximately the same. Both the positive and negative examples have been annotated with the same 26 genomic and epigenomic features used for the original Mendelian dataset. We trained hyperSMURF and parSMURF on the overall original Mendelian dataset, and then we tested the resulting models on the unseen separated new Mendelian dataset used for validation. Because the new positive set also contains small insertions and deletions, similarly to [29], to predict the pathogenicity of the deletions, we used the maximum score of any nucleotide included in the deleted sequence, while for insertions we used the maximum score computed for the 2 nucleotides that surround the insertion. Results with the independent Mendelian test set show that the models are able to obtain relatively high AUPRC results, especially when parSMURF is applied, showing that our models can nicely generalize. Also with this new independent dataset parSMURF significantly outperforms hyperSMURF (Table 5).

**Table 5:** hyperSMURF and parSMURF (with grid search and Bayesian optimization) prediction performance obtained over a fully independent Mendelian test set composed of newly annotated pathogenic variants (positive examples) and common neutral variants (negative examples)

Model	AUROC	AUPRC
hyperSMURF	0.945216	0.098153
parSMURF, grid search	0.941026	0.409067
parSMURF, Bayesian optimizaion	0.928158	0.192568

**Table 6:** Examples of pathogenic Mendelian variants better ranked by parSMURF with respect to hyperSMURF

chr	pos	hS rank $- \mathcal {H}_g$ rank	hS rank $- \mathcal {H}_b$ rank
1	100,661,453	2,308,597	169,786
3	12,421,189	663,054	421,027
X	138,612,889	194,290	111,499
13	100,638,902	70,175	69,069
6	118,869,423	63,078	55,789
16	31,202,818	50,539	103,623
12	121,416,444	21,848	65,773

The first 2 cols report the chromosomal coordinates, while the last 2 the difference in ranking between, respectively, parSMURF with grid search ($\mathcal{H}_g$) and with Bayesian optimizer ($\mathcal{H}_b$) with respect to hyperSMURF. The larger the absolute difference, the greater the improvement (see also Supplementary Table S6 for more information).

**Table 7:** Summary of performance improvements obtained by parSMURF by tuning the learning parameters on the Mendelian dataset

Parameters	Training set mean (SD)		Test set mean (SD)	
	AUROC	AUPRC	AUROC	AUPRC
Default parameters	0.99958 (0.00005)	0.53143 (0.02714)	0.99281 (0.00032)	0.42332 (0.00391)
Grid search	0.99986 (0.00009)	0.60023 (0.15977)	0.98968 (0.00140)	0.47025 (0.00585)
Bayesian optimizer	0.99989 (0.00011)	0.65388 (0.22123)	0.99264 (0.00043)	0.46153 (0.00302)

Results are averaged across 10 repetitions of the 10-fold cytoband-aware CV. AUROC and AUPRC are averaged across the 10 folds; standard deviation (SD) in parentheses. Default parameters: nParts 100, fp 2, ratio 3, nTrees 10, mTry 5.

#### Improving predictions of GWAS hits

A similar experimental set-up has been used for improving the predictions of GWAS hits. At first we executed parSMURF with the default parameters as reference for the next batches of experiments. Then, we tested the auto-tuning feature by performing a grid search over the hyper-parameter space $\mathcal {H}_g$ defined in Table 8, col. 2. Such hyperspace provides 256 possible hyper-parameter combinations $h \in \mathcal {H}_g$. Next, we tested the BO by defining the hyper-parameter space $\mathcal {H}_b$ as in Table 8, col. 3.

**Table 8:** Hyper-parameter space for grid search and Bayesian optimization used for auto-tuning parSMURF on the GWAS dataset

Parameter	Grid search	Bayesian optimization
nParts	{10, 20, 30, 40}	[10, 40]
fp	{1, 2, 5, 10}	[1, 10]
ratio	{1, 2, 5, 10}	[1, 10]
k	{5}	{5}
nTrees	{10, 20, 50, 100}	[10, 100]
mTry	{30}	{30}

Results are reported in Table 9. As for the Mendelian dataset, AUROC is very high in all experiments. On the other hand, test results show a significant increase of AUPRC with both auto-tuning strategies, with the grid search leading to a better outcome than the BO. Supplementary Figs S3 and S4 show the ROC and precision-recall curves of hyperSMURF and parSMURF.

**Table 9:** Summary of the performance improvements obtained by parSMURF by tuning its learning parameters with the GWAS Catalog dataset

Parameters	AUROC mean (SD)	AUPRC mean (SD)
Default parameters	0.99426 (0.00169)	0.48058 (0.07138)
Grid search	0.99459 (0.00174)	0.72533 (0.03616)
Bayesian optimizer	0.99346 (0.00193)	0.71945 (0.03675)

Results are averaged across 10 repetitions of the 10-fold cytoband-aware cross-validation. AUROC and AUPRC are averaged across the 10 folds. Default parameters: nParts 100, fp 2, ratio 3, nTrees 10, mTry 5.

These results further show that fine tuning of learning parameters is fundamental to significantly improving prediction performances, showing also that parSMURF is a useful tool to automatically find “near-optimal” learning parameters for the prediction task under study.

#### Assessment of the effect on prediction performance of the variants imbalance across regulatory regions

As recently pointed out by Caron et al. [28], pathogenic scores predicted by several state-of-the-art methods are biased towards some specific regulatory region types. Indeed also with Mendelian and GWAS data the positive set of variants is located in different functional non-coding regions (e.g., 5′ UTR, 3′ UTR, or promoter) and is not evenly distributed over them. This is also the case for the negative set (see Supplementary Tables S4 and S5). Because of this imbalance, performance in different categories is different as already mentioned by Smedley et al. for the ReMM score on the Mendelian data [29]. It is possible that our parSMURF parameter optimization will favour different categories because of the number of available positives and the different imbalance between positives and negatives across different genomic regions. To show that the optimization is robust to this characteristic of the data we compared performances on each genomic category before and after parameter optimization. Variant categories have been defined through Jannovar [54] using the RefSeq database.

Then we retrained and re-optimized the parameters on a training set using cytoband-aware CV, where all categories have the same imbalance by subsampling negatives to the smallest imbalance of the categories. In more detail, we used the following strategy: (i) subsample the negatives to the same imbalance in all categories. Mark the variant if it is in this new subset; (ii) partition the whole dataset into 10 folds as done previously; (iii) for each training step select only the previously marked variants of the 9 training folds; (iv) subsample the test set using the same categorization and ratios as in (i). To take into account the variability between runs, we repeated this process 10 times for the Mendelian dataset and 5 times for the GWAS dataset. Using this strategy both training and test sets are equally “per region balanced,” so that category unbalance is kept under control and we can correctly evaluate whether our approach may unnaturally inflate predictions towards a specific region owing to the original per-region imbalance of both datasets.

Results are shown in Supplementary Figs S5 and S6: for all variant categories we see a performance gain or similar performance in parSMURF with respect to hyperSMURF for both the Mendelian and GWAS dataset, suggesting that parSMURF is robust to the categorical composition of the variants. Moreover in the “per region balanced” setting AUPRC results are systematically better with the Mendelian dataset (Supplementary Fig. S5) and always better than or comparable to the GWAS data (Supplementary Fig. S6). These experimental results show that both hyperSMURF and parSMURF can properly handle different imbalances of variant categories, by using ”smart” balancing techniques on the training set able to both balance and at the same time maintain a large coverage of the available training data. The increase of performance of parSMURF with respect to hyperSMURF is not driven by the under- or overrepresentation of variants belonging to a particular region type but by its capacity of automatically fine tuning the set of its hyper-parameters, according to the given task at hand.

#### Analysis of the hyper-parameters

Because we adopted CV techniques to estimate the generalization performance of the models, we averaged the best parameter values separately estimated for each fold, in order to obtain a single set of optimal parameters. Tables S1 and S2 of the Supplementary Information show the sets of best hyper-parameters found by both the optimization techniques with the Mendelian and GWAS datasets.

Of the 6 hyper-parameters, we noticed that nParts, fp, and ratio are the main factors that drive the performance improvement. The fp and ratio hyper-parameters provide the rebalancing of the classes. A larger fp value translates into a larger number of positive examples generated through the SMOTE algorithm (see Methods), thus reducing the imbalance between positive and negative examples in the training set: Supplementary Tables S1 and S2 show that by enlarging the ratio of novel positive examples parSMURF improves results over hyperSMURF, and confirm that fine-tuned balancing techniques can improve the results.

The ratio hyper-parameter controls the ratio between negative and positive examples of the training set. Results in Tables S1 and S2 show that values larger than the default ones improve performance, because in this way we can both reduce the imbalance between negatives and positives (for the Mendelian datasets we move from 36,000:1 to 10:1, and for GWAS from 700:1 to 10:1), and at the same time we maintain a relatively large coverage of the negative data (in each partition negative examples are sampled in such a way to obtain 10 negatives for each positive of the training set).

The results also show that a larger coverage of negative examples is obtained by incrementing the nParts hyper-parameter, because by increasing the number of partitions, fewer negatives are discarded. Moreover more random forests are trained, thus improving the generalization capabilities of the hyper-ensemble. Finally, for the GWAS dataset, the mTry hyper-parameter plays a fundamental role in the increment of the performance, due to the high number of features of the dataset. Overall, the analysis of the hyper-parameters confirms that their fine tuning is fundamental to improving the performance of the hyper-ensemble.

## Conclusion

In this article we present parSMURF, a high-performance computing tool for the prediction of pathogenic variants, designed to deal with the issues related to the inference of accurate predictions with highly unbalanced datasets. We showed that hyperSMURF, despite its encouraging results with different genomic datasets, is hindered by 2 major drawbacks: a very demanding computing time and the need of a proper fine tuning of the learning parameters. The proposed parSMURF method provides a solution for both problems, through 2 efficient parallel implementations—parSMURF^1^ and parSMURF^n^—that scale well with, respectively, multi-core machines and multi-node HPC cluster environments.

Results with synthetic datasets show that parSMURF scales nicely with large datasets, introducing a sensible speed-up with respect to the pure sequential version. Especially for large datasets, as expected, we should prefer the hybrid MPI-multi-thread version parSMURF^n^, while for relatively smaller datasets we can obtain a reasonable speed-up also with the pure multi-thread version parSMURF^1^ that can run also with an off-the-shelf laptop or desktop computer, by exploiting the multi-core architecture of modern computers.

parSMURF features 2 different strategies for the auto-tuning of the learning parameters, both of them effective: the first is based on an exhaustive grid search, which proves to be effective in finding the best combination of hyper-parameters in terms of maximizing the AUPRC rating but turns out to be very computing-intensive. The other strategy is Bayesian optimization–based and aims to find a near-optimal hyper-parameter combination in a fraction of the time compared to the grid search strategy. Experimental results with Mendelian diseases and GWAS hits in non-coding regulatory regions show that parSMURF can enhance hyperSMURF performance, confirming that fine tuning of learning hyper-parameters may lead to significant improvements of the results.

The high level of parallelism of parSMURF, its auto-tuning hyper-parameters capabilities, and its easy-to-use software interface allow the user to apply this tool to ranking and classification problems characterized by highly imbalanced big data. This situation commonly arises in genomic medicine problems because only a small set of “positive” examples is usually available to train the learning machines. For this reason parSMURF can be a useful tool not only for the prediction of pathogenic variants but also for any imbalanced ranking and classification problem in genomic medicine, provided that suitable big data are available for the problem at hand.

## Availability of Source Code and Requirements

Project name: parSMURF

Project home page: https://github.com/AnacletoLAB/parSMURF

RRID:SCR_017560

Operating system(s): Linux

Programming language: C++, Python 2.7

Requirements for parSMURF^1^: Multi-core x86-64 processor, 512 MB RAM, C++ compiler supporting OpenMP standard.

Requirements for parSMURF^n^: Multi-core x86-64 processor, 1,024 MB RAM, implementation of MPI library (i.e., OpenMPI or IntelMPI) installed on each node of the cluster, a reasonably fast interconnecting infrastructure.

License: GNU General Public License v3

## Availability of Supporting Data and Materials

Datasets used for the assessment of scalability and prediction quality are available via the Open Science Foundation project [55]. Supporting data are available at GigaDB data repository [56].

## Additional Files

**Supplementary Figure S**1. Plot of Receiver Operating Characteristic curve of the predictions for the Mendelian dataset using 3 sets of hyper-parameters.

**Supplementary Figure S**2. Plot of Precision-Recall curve of the predictions for the Mendelian dataset using 3 sets of hyper-parameters.

**Supplementary Figure S**3. Plot of Receiver Operating Characteristic curve of the predictions for the GWAS dataset using 3 sets of hyper-parameters.

**Supplementary Figure S**4. Plot of Precision-Recall curve of the predictions for the GWAS dataset using 3 sets of hyper-parameters.

**Supplementary Figure S**5. Prediction performances (AUROC and AUPRC) of HyperSMURF and parSMURF for the Mendelian dataset, with both the Original imbalanced Mendelian data set and with the separated “per-region balanced” Mendelian data.

**Supplementary Figure S**6. Prediction performances (AUROC and AUPRC) of HyperSMURF and parSMURF for the GWAS dataset, with both the Original imbalanced GWAS data set and with the separated “per-region balanced” GWAS data.

**Supplementary Table S**1. Optimal sets of hyper-parameters returned by the optimizers embedded in parSMURF while training the model with the Mendelian dataset.

**Supplementary Table S**2. Optimal sets of hyper-parameters returned by the optimizers embedded in parSMURF while training the model with the GWAS dataset.

**Supplementary Table S3**. Spearman correlation between HyperSMURF and parSMURF scores for each of the 26 features of the Mendelian dataset.

**Supplementary Table S4**. Imbalance of the number of negative and positive examples across different regulatory region types in the Mendelian dataset.

**Supplementary Table S5**. Imbalance of the number of negative and positive examples across different regulatory region types in the GWAS dataset.

**Supplementary Table S6**. Examples of pathogenic Mendelian single nucleotide variants where parSMURF sensibly outperformed hyperSMURF.

**Supplementary Table S7**. List of newly annotated pathogenic variants used as independent test set to assess the generalization capabilities of parSMURF.

giaa052_GIGA-D-19-00126_Original_Submission

giaa052_GIGA-D-19-00126_Revision_1

giaa052_GIGA-D-19-00126_Revision_2

giaa052_GIGA-D-19-00126_Revision_3

giaa052_GIGA-D-19-00126_Revision_4

giaa052_Response_to_Reviewer_Comments_Original_Submission

giaa052_Response_to_Reviewer_Comments_Revision_1

giaa052_Response_to_Reviewer_Comments_Revision_2

giaa052_Response_to_Reviewer_Comments_Revision_3

giaa052_Reviewer_1_Report_Original_Submission Maria Chikina -- 5/28/2019 Reviewed

giaa052_Reviewer_2_Report_Original_Submission Antonio Rausell -- 6/27/2019 Reviewed

giaa052_Reviewer_2_Report_Revision_1 Antonio Rausell -- 1/21/2020 Reviewed

giaa052_Reviewer_2_Report_Revision_2 Antonio Rausell -- 4/9/2020 Reviewed

giaa052_Supplemental_Figures_and_Tables

## Abbreviations

AUPRC: area under the precision-recall curve; AUROC: area under the receiver operating characteristic curve; CADD: Combined Annotation-Dependent Depletion; CV: cross-validation; FATHMM-MKL: Functional Analysis through Hidden Markov Models and Multiple Kernel Learning; G/C: guanine-cytosine; gkm-SVM: Gapped k-mer Support Vector Machine; GWAS: genome-wide association study; GWAVA: Genome-Wide Annotation of Variants; MPI: Message Passing Interface; NCBI: National Center for Biotechnology Information; NGS: next-generation sequencing; OpenMP: Open Multi-Processing; RAM: random access memory; SLURM: Simple Linux Utility for Resource Management; SMOTE: Synthetic Minority Over-sampling Technique; SNV: single-nucleotide variant; UTR: untranslated region.

## Competing Interests

The authors declare that they have no competing interests.

## Funding

A.P. thanks Università degli Studi di Milano for funding this publication through its special funds for Application Processing Charges. G.V. thanks CINECA and Regione Lombardia for supporting the projects “HyperGeV :Detection of Deleterious Genetic Variation through Hyper-ensemble Methods” and “HPC-SoMuC: Development of Innovative HPC Methods for the Detection of Somatic Mutations in Cancer.” P.N.R. received support from the National Institutes of Health (NIH), Monarch Initiative (OD #5R24OD011883). G.G., M.M., M.R, and G.V. received support from the Università degli Studi di Milano, project number 15983, titled “Discovering Patterns in Multi-Dimensional Data.” G.V., A.P., M.S., and M.R. received support form the MIUR-DAAD Joint Mobility Program “Developing machine learning methods for the prioritization of regulatory variants in human disease,” Prog. n. 33122.

## Authors' Contributions

Conceptualization and Methodology: A.P., G.V. Formal Analysis: A.P., G.V., G.G., M.F. Data Curation and Investigation: M.S., M.R., D.D. Software: A.P., G.G., M.F., and L.C. Supervision: G.V., P.R. Validation: A.P., T.C. Funding Acquisition: G.G., M.M., G.V. Writing - Original Draft Preparation: G.V., A.P., M.M. Writing - Review & Editing: all authors.
