# Supplementary material for: parSMURF, a high-performance computing tool for the genome-wide detection of pathogenic variants
Source: Gigascience. 2020 May 23;9(5):giaa052. doi: 10.1093/gigascience/giaa052 (PMC7244787; doi:10.1093/gigascience/giaa052)
Supplement: giaa052_Supplemental_Figures_and_Tables [file giaa052_supplemental_figures_and_tables.pdf]

GigaScience, 2019, 1–9

doi: xx.xxxx/xxxx
Manuscript in Preparation
Supplementary material

SUPPLEMENTARY MATERIAL

parSMURF, a High Performance Computing tool for the genome-wide detection of pathogenic variants – Supplementary Information

Alessandro Petrini[1], Marco Mesiti[1], Max Schubach[2,3], Marco Frasca[1], Daniel Danis[4], Matteo Re[1], Giuliano Grossi[1], Luca Cappelletti[1], Tiziana Castrignanò[5], Peter N. Robinson[4] and Giorgio Valentini[1,6]

[1]AnacletoLab – Dipartimento di Informatica, Università degli Studi di Milano, Italy and [2]Berlin Institute of Health (BIH), Berlin, Germany and [3]Charité – Universitätsmedizin Berlin, Berlin, Germany and [4]The Jackson Laboratory for Genomic Medicine, Farmington CT, USA and [5]CINECA, SCAI SuperComputing Applications and Innovation Department, Roma, Italy and [6]CINI National Laboratory in Artificial Intelligence and Intelligent Systems – AIIS, Roma, Italy

List of Figures

1 Plot of Receiver Operating Characteristic curve of the predictions for the Mendelian dataset using 3 sets of hyper-parameters: default (blue), best from the grid search (red) and best from the Bayesian optimizer (green) 3

2 Plot of Precision-Recall curve of the predictions for the Mendelian dataset using 3 sets of hyper-parameters: default (blue), best from the grid search (red) and best from the Bayesian optimizer (green) 3

3 Plot of Receiver Operating Characteristic curve of the predictions for the GWASn dataset using 3 sets of hyper-parameters: default (blue), best from the grid search (red) and best from the Bayesian optimizer (green) 3

4 Plot of Precision-Recall curve of the predictions for the GWAS dataset using 3 sets of hyper-parameters: default (blue), best from the grid search (red) and best from the Bayesian optimizer (green) 3

5 Prediction performances (AUROC and AUPRC) for the Mendelian dataset, with both the Original imbalanced Mendelian data set and with the separated "per-region balanced" Mendelian data used for validation using *hyperSMURF* (hS) and *parSMURF* (pS) with Bayesian optimization hyper-ensembles (set of hyper-parameters found by the Bayesian optimizer: `nParts`: 36 - `fp`: 3 - `ratio`: 5 - `k`: 5 - `numTrees`: 55 - `mtry`: 5). Each group shows prediction performances divided by regulatory region type. "Global" group shows prediction performances of the entire dataset, disregarding the regulatory region type. AUROC and AUPRC have been averaged across "cytoband-aware" 10-fold cross validation. Results have been averaged across 10 repetitions of the same experimental setup (error bars represent standard deviation). "Original imbalanced" and "Per-region balanced" stands respectively for the original imbalanced setting and the balanced setting where the same ratio of positives versus negatives in both training and test sets is maintained in each regulatory region. 4

6 Prediction performances (AUROC and AUPRC) for the GWAS dataset, with both the Original imbalanced GWAS data set and with the separated "per-region balanced" GWAS data used for validation using *hyperSMURF* (hS) and *parSMURF* (pS) with Bayesian optimization hyper-ensembles (set of hyper-parameters found by the Bayesian optimizer: `nParts`: 10 - `fp`: 2 - `ratio`: 5 - `k`: 5 - `numTrees`: 50 - `mtry`: 30). Each group shows prediction performances divided by regulatory region type. "Global" group shows prediction performances of the entire dataset, disregarding the regulatory region type. AUROC and AUPRC have been averaged across "cytoband-aware" 10-fold cross validation. Results have been averaged across 5 repetitions of the same experimental setup (error bars represent standard deviation). "Original imbalanced" and "Per-region balanced" stands respectively for the original imbalanced setting and the balanced setting where the same ratio of positives versus negatives in both training and test sets is maintained in each regulatory region. . . . 5

List of Tables

1 Optimal sets of hyper-parameters returned by the optimizers embedded in *parSMURF* while training the model with the Mendelian dataset. Refer to Table 1 in the main paper for a brief explanation of each parameter. Default values are those used with *hyperSMURF*. As Grid and Bayesian optimizer returns the set of best hyper-parameters for each fold of the cross-validation, optimal values have been computed by averaging each parameter over the folds. 6

2 Optimal sets of hyper-parameters returned by the optimizers embedded in *parSMURF* while training the model with the GWAS dataset. Refer to Table 1 in the main paper for a brief explanation of each parameter. Default values are those used with *hyperSMURF*. As Grid and Bayesian optimizer returns the set of best hyper-parameters for each fold of the cross-validation, optimal values have been computed by averaging each parameter over the folds. 6

3 Spearman correlation between HyperSMURF and parSMURF scores for each of the 26 features of the Mendelian dataset. 6

4 Imbalance of the number of negative and positive examples across different regulatory region types in the Mendelian dataset. In column 4, the ratio between negative and positive samples; in bold, the lowest ratio used to under-sample the negatives for each regulatory region. The last column reports the number of down-sampled negatives for each regulatory region after applying the best ratio found in column 4. 6

5 Imbalance of the number of negative and positive samples across different regulatory region types in the GWAS dataset. In column 4, the ratio between negative and positive samples; in bold, the lowest ratio used to under-sample the negatives for each regulatory region. The last column reports the number of down-sampled negatives for each regulatory region after applying the best ratio found in column 4. 7

6 Examples of pathogenic Mendelian single nucleotide variants where *parSMURF* sensibly outperformed *hyperSMURF*. The first two columns refer to the chromosomal coordinates of the variant. Ref and Alt to the reference and alternative allele; OMIM to the OMIM code of the associated Mendelian disease; Gene to the symbolic name of the target gene; PMID to the PubMed ID of the related publication; Region to the type of the regulatory region. The two last columns refer to the difference of ranking between respectively *parSMURF* with grid optimization and *parSMURF* with Bayesian optimization with respect to *hyperSMURF*. 7

7 List of newly annotated pathogenic variants used as independent test set to assess the generalization capabilities of *parSMURF*. The first two columns refer to chromosomal coordinates, REF and ALT to the reference and alternative allele (including both SNVs, micro-insertions and micro-deletions), Region the type of regulatory region, Gene the symbolic name of the gene and PMID the PubMed ID of the related publication. 8

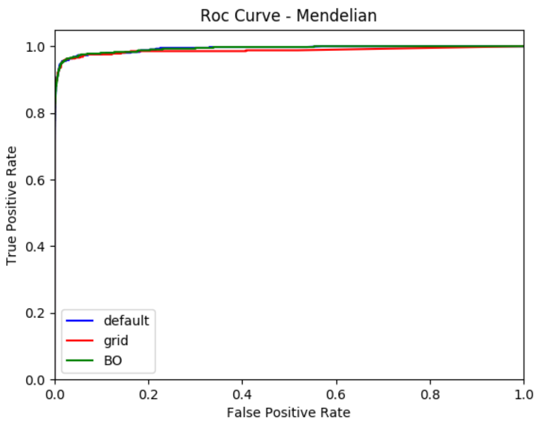

Figure 1. Plot of Receiver Operating Characteristic curve of the predictions for the Mendelian dataset using 3 sets of hyper-parameters: default (blue), best from the grid search (red) and best from the Bayesian optimizer (green)

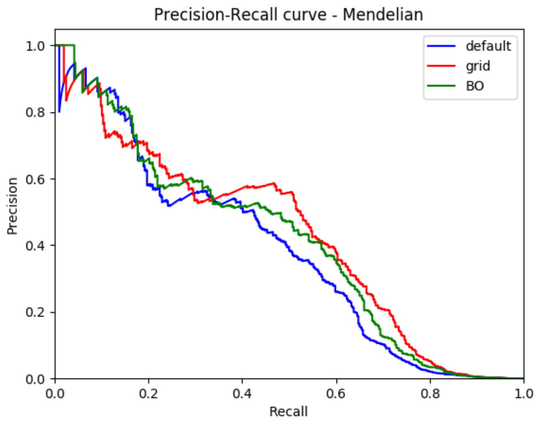

Figure 2. Plot of Precision-Recall curve of the predictions for the Mendelian dataset using 3 sets of hyper-parameters: default (blue), best from the grid search (red) and best from the Bayesian optimizer (green)

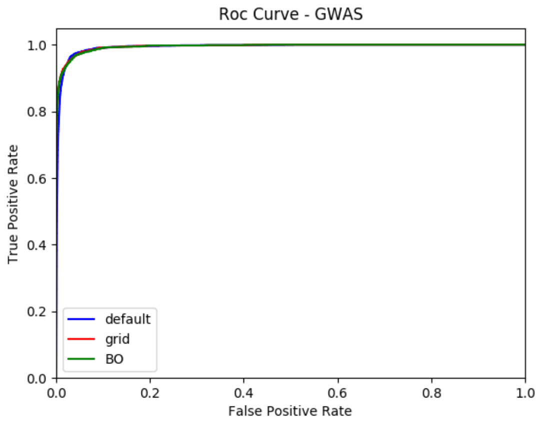

Figure 3. Plot of Receiver Operating Characteristic curve of the predictions for the GWASn dataset using 3 sets of hyper-parameters: default (blue), best from the grid search (red) and best from the Bayesian optimizer (green)

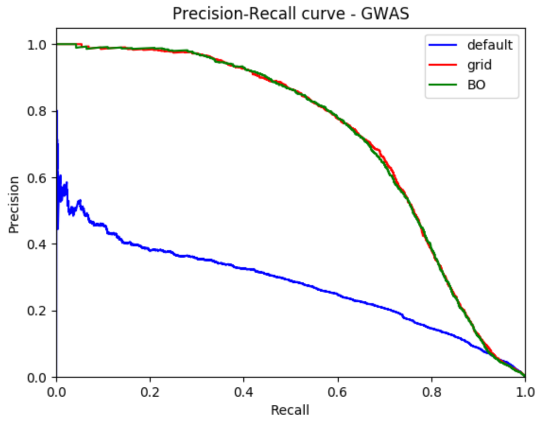

Figure 4. Plot of Precision-Recall curve of the predictions for the GWAS dataset using 3 sets of hyper-parameters: default (blue), best from the grid search (red) and best from the Bayesian optimizer (green)

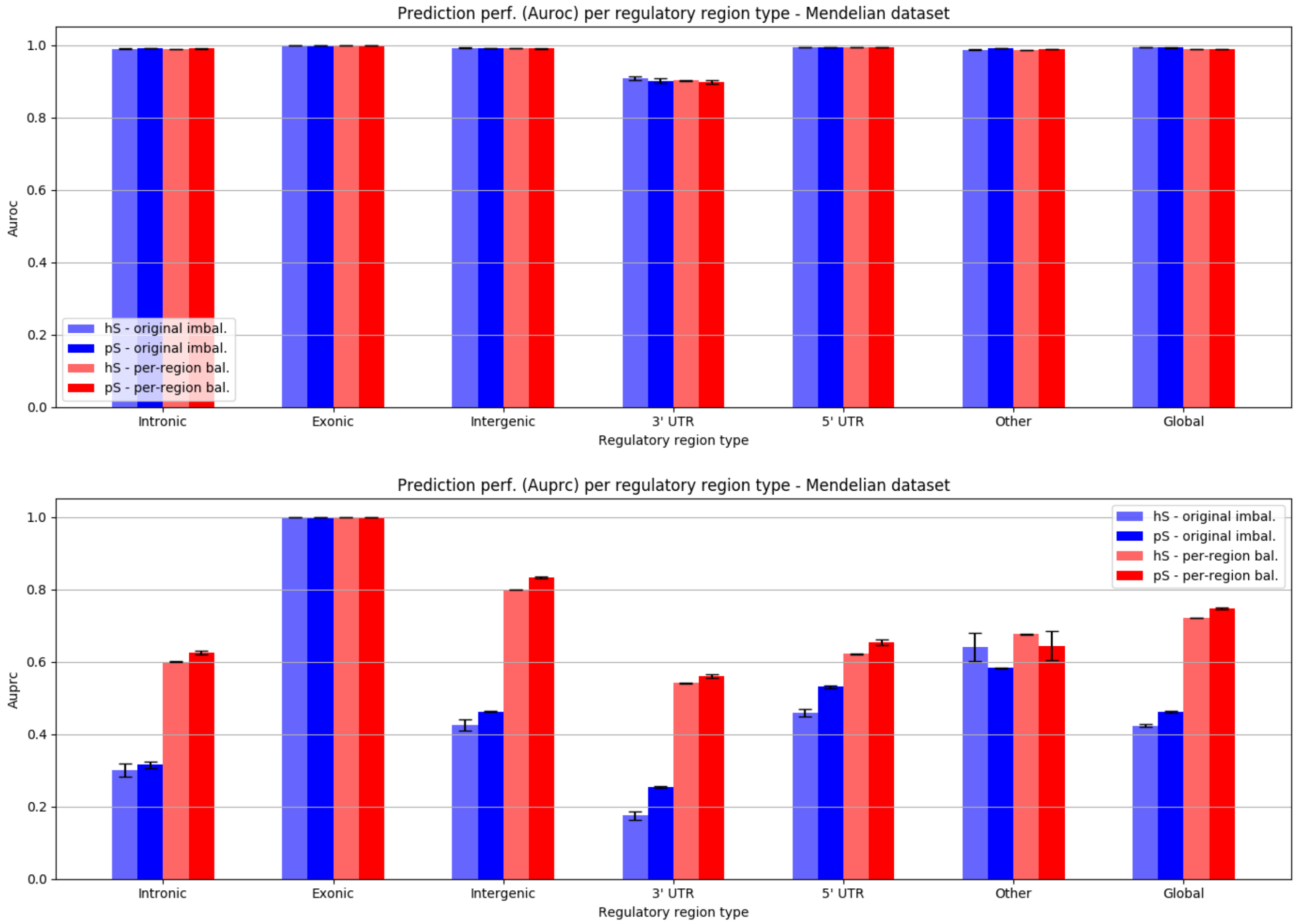

Figure 5. Prediction performances (AUROC and AUPRC) for the Mendelian dataset, with both the Original imbalanced Mendelian data set and with the separated "per-region balanced" Mendelian data used for validation using *hyperSMURF* (hS) and *parSMURF* (pS) with Bayesian optimization hyper-ensembles (set of hyper-parameters found by the Bayesian optimizer: `nParts`: 36 - `fp`: 3 - `ratio`: 5 - `k`: 5 - `numTrees`: 55 - `mtry`: 5). Each group shows prediction performances divided by regulatory region type. "Global" group shows prediction performances of the entire dataset, disregarding the regulatory region type. AUROC and AUPRC have been averaged across "cytoband-aware" 10-fold cross validation. Results have been averaged across 10 repetitions of the same experimental setup (error bars represent standard deviation). "Original imbalanced" and "Per-region balanced" stands respectively for the original imbalanced setting and the balanced setting where the same ratio of positives versus negatives in both training and test sets is maintained in each regulatory region.

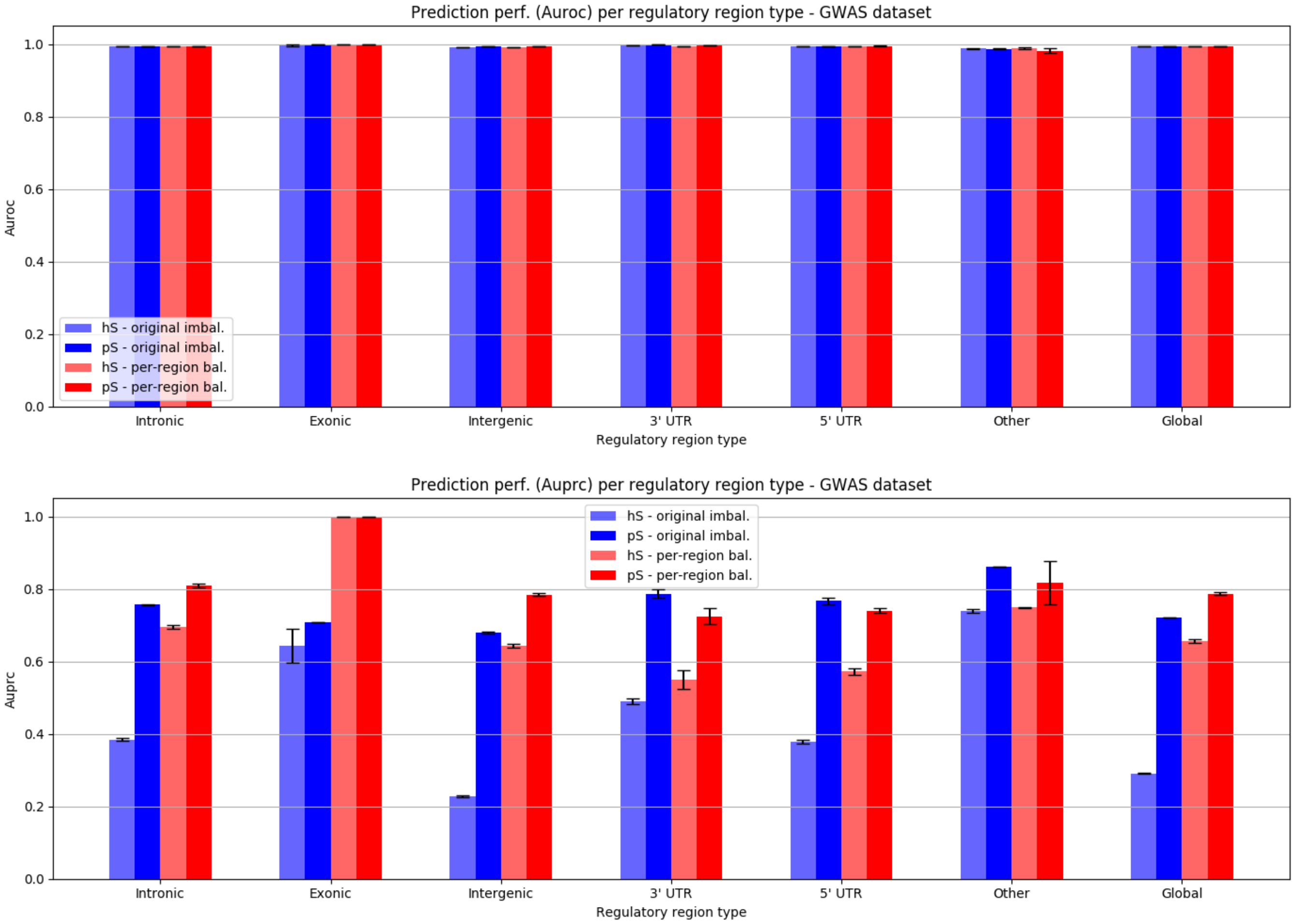

Figure 6. Prediction performances (AUROC and AUPRC) for the GWAS dataset, with both the Original imbalanced GWAS data set and with the separated "per-region balanced" GWAS data used for validation using *hyperSMURF* (hS) and *parSMURF* (pS) with Bayesian optimization hyper-ensembles (set of hyper-parameters found by the Bayesian optimizer: `nParts`: 10 - `fp`: 2 - `ratio`: 5 - `k`: 5 - `numTrees`: 50 - `mtry`: 30). Each group shows prediction performances divided by regulatory region type. "Global" group shows prediction performances of the entire dataset, disregarding the regulatory region type. AUROC and AUPRC have been averaged across "cytoband-aware" 10-fold cross validation. Results have been averaged across 5 repetitions of the same experimental setup (error bars represent standard deviation). "Original imbalanced" and "Per-region balanced" stands respectively for the original imbalanced setting and the balanced setting where the same ratio of positives versus negatives in both training and test sets is maintained in each regulatory region.

Table 1. Optimal sets of hyper-parameters returned by the optimizers embedded in *parSMURF* while training the model with the Mendelian dataset. Refer to Table 1 in the main paper for a brief explanation of each parameter. Default values are those used with *hyperSMURF*. As Grid and Bayesian optimizer returns the set of best hyper-parameters for each fold of the cross-validation, optimal values have been computed by averaging each parameter over the folds.

	nParts	fp	ratio	k	nTrees	mtry
Default	100	2	3	5	10	5
Grid search	260	7	10	5	50	4
Bayesian optimizer	74	8	10	5	50	5

Table 2. Optimal sets of hyper-parameters returned by the optimizers embedded in *parSMURF* while training the model with the GWAS dataset. Refer to Table 1 in the main paper for a brief explanation of each parameter. Default values are those used with *hyperSMURF*. As Grid and Bayesian optimizer returns the set of best hyper-parameters for each fold of the cross-validation, optimal values have been computed by averaging each parameter over the folds.

	nParts	fp	ratio	k	nTrees	mtry
Default	100	2	3	5	10	5
Grid search	10	5	10	5	100	30
Bayesian optimizer	10	4	10	5	100	30

Table 3. Spearman correlation between HyperSMURF and parSMURF scores for each of the 26 features of the Mendelian dataset.

Feature name	HyperSMURF	parSMURF default	parSMURF grid	parSMURF BO
CpGobsExp	0.13968	0.13819	0.13893	0.13132
CpGperCpG	0.13969	0.13819	0.13893	0.13132
CpGperGC	0.13968	0.13819	0.13893	0.13132
DGVCount	0.03413	0.04442	0.05647	0.06046
DnaseClusteredHyp	0.32951	0.32044	0.24895	0.28336
DnaseClusteredScore	0.32908	0.32009	0.24869	0.28315
EncH3K27Ac	0.30594	0.30151	0.24630	0.30958
EncH3K4Me1	0.31055	0.30229	0.25376	0.31746
EncH3K4Me3	0.23845	0.23367	0.19111	0.22128
GCContent	0.20083	0.18597	0.15363	0.19790
GerpRS	0.35444	0.35057	0.31584	0.30759
GerpRSpv	0.18839	0.18847	0.15510	0.15381
ISCApath	0.00046	0.00916	−0.01227	0.00711
commonVar	−0.18408	−0.20035	−0.13659	−0.16558
dbVARCount	0.03413	0.04442	0.05647	0.06046
fantom5Perm	0.06291	0.06130	0.05101	0.05391
fantom5Robust	0.06436	0.06269	0.05217	0.05513
fracRareCommon	0.18258	0.20198	0.15003	0.17902
mamPhastCons46way	0.14808	0.14348	0.15451	0.16040
mamPhyloP46way	0.37928	0.38087	0.37977	0.43174
numTFBSConserved	0.13326	0.14405	0.14100	0.13788
priPhastCons46way	0.24858	0.25781	0.26809	0.29352
priPhyloP46way	0.33472	0.33576	0.33014	0.37434
rareVar	0.02916	0.03668	0.05208	0.05687
verPhastCons46way	0.15317	0.15002	0.16128	0.16888
verPhyloP46way	0.38423	0.38685	0.38594	0.43717

Table 4. Imbalance of the number of negative and positive examples across different regulatory region types in the Mendelian dataset. In column 4, the ratio between negative and positive samples; in bold, the lowest ratio used to under-sample the negatives for each regulatory region. The last column reports the number of down-sampled negatives for each regulatory region after applying the best ratio found in column 4.

Category	Number of positive sampl.	Number of negative sampl.	Ratio (neg/pos)	Downsampled negative samples
Intronic	75	5393802	71917	64625
Exonic non coding	2	35058	17529	1723
Intergenic	126	8325494	66075	108570
3' UTR	30	144348	4811	25850
5' UTR	158	843572	5339	136143
Other	15	12925	**861**	12925
			**Tot. negatives:**	349836

Table 5. Imbalance of the number of negative and positive samples across different regulatory region types in the GWAS dataset. In column 4, the ratio between negative and positive samples; in bold, the lowest ratio used to under-sample the negatives for each regulatory region. The last column reports the number of down-sampled negatives for each regulatory region after applying the best ratio found in column 4.

Category	Number of positive sampl.	Number of negative sampl.	Ratio (neg/pos)	Downsampled negative samples
Intronic	937	539311	575	115166
Exonic non coding	2	3511	1755	245
Intergenic	891	832571	934	109512
3’ UTR	46	14470	314	5653
5’ UTR	228	84300	370	28023
Other	11	1352	**122**	1352
			**Tot. negatives:**	259951

Table 6. Examples of pathogenic Mendelian single nucleotide variants where *parSMURF* sensibly outperformed *hyperSMURF*. The first two columns refer to the chromosomal coordinates of the variant. Ref and Alt to the reference and alternative allele; OMIM to the OMIM code of the associated Mendelian disease; Gene to the symbolic name of the target gene; PMID to the PubMed ID of the related publication; Region to the type of the regulatory region. The two last columns refer to the difference of ranking between respectively *parSMURF* with grid optimization and *parSMURF* with Bayesian optimization with respect to *hyperSMURF*.

Chr	Position	Ref	Alt	OMIM	Gene	PMID	Region	Rank diff. Grid/Default	Rank diff. BO/Default
chr1	100661453	T	G	MIM 248600	DBT	20570198 [1]	3’ UTR	2308597	169786
chr3	12421189	A	G	MIM 604367	PPARG	15531525 [2]	Promoter	663054	421027
chrX	138612889	G	A	MIM 306900	F9	23472758 [3]	Promoter	194290	111499
chr13	100638902	A	G	MIM 609637	ZIC2	22859937 [4]	3’ UTR	70175	69069
chr6	118869423	A	G	MIM 609909	PLN	18241046 [5]	Promoter	63078	55789
chr16	31202818	G	A	MIM 608030	FUS	23847048 [6]	3’ UTR	50539	103623
chr12	121416444	T	G	MIM 600496	HNF1A	22413961 [7]	Promoter	21848	65773

Table 7. List of newly annotated pathogenic variants used as independent test set to assess the generalization capabilities of *parSMURF*. The first two columns refer to chromosomal coordinates, REF and ALT to the reference and alternative allele (including both SNVs, micro-insertions and micro-deletions), Region the type of regulatory region, Gene the symbolic name of the gene and PMID the PubMed ID of the related publication.

Chr	Pos	Ref	Alt	Region	Gene	PMID
1	155261709	G	A	exonic non-coding	PKLR	18708292
1	155263324	C	T	exonic non-coding	PKLR	26728349
1	155271259	C	G	promoter	PKLR	18708292
1	155271269	CAGAGA	C	promoter	PKLR	26728349
3	10183453	AGCGCGCACGCAGCTCCGCCCC GCGTCCGACCCGCGGATCCCGCGGC	A	5' UTR	VHL	30006056
3	10183466	C	CTCCGCCCCGCG	5' UTR	VHL	30006056
3	38675715	C	T	promoter	SCN5A	27625342
4	89444948	C	T	promoter	PIGY	26293662
5	131705516	G	A	5' UTR	SLC22A5	31187905
6	100040906	G	T	enhancer	DHS6S1	26507665
6	100040987	G	C	enhancer	DHS6S1	27551809
6	100041040	C	T	enhancer	DHS6S1	26507665
7	19157199	C	T	5' UTR	TWIST1	30040876
7	19157207	G	T	5' UTR	TWIST1	30040876
7	156584142	A	T	enhancer	LMBR1	27592358
7	156585476	C	G	enhancer	SHH	29543231
8	11565816	G	C	5' UTR	GATA4	25099673
8	19796936	A	G	5' UTR	LPL	27578109
8	102505149	GA	G	5' UTR	GRHL2	29499165
8	102505272	CT	C	5' UTR	GRHL2	29499165
8	102505561	G	T	5' UTR	GRHL2	29499165
9	21974830	GCTCCCCGCCGCCCGCTGCCTGCTC	G	5' UTR	CDKN2A	26581427
9	21974847	G	A	5' UTR	CDKN2A	26581427
9	21974868	A	T	5' UTR	CDKN2A	26581427
9	21974916	CCCT	C	5' UTR	CDKN2A	26581427
9	21975024	GAGT	AAAG	5' UTR	CDKN2A	29216274
10	23481888	AGCGGCGGCTGCGGCGGCGCGGCGCCG	A	exonic non-coding	PTF1A	28663161
10	23508442	A	G	enhancer	PTF1A	28663161
11	31828391	ACTT	CA	5' UTR	PAX6	30291432
11	31828391	AC	A	5' UTR	PAX6	30291432
11	31828396	C	T	5' UTR	PAX6	30291432
11	31828461	TAA	T	5' UTR	PAX6	30291432
11	31828474	CT	C	5' UTR	PAX6	30291432
11	31832375	C	T	5' UTR	PAX6	30291432
11	67250359	CCG	C	promoter	AIP	20506337
13	110802675	G	T	3' UTR	COL4A1	27666438
13	110802678	C	T	3' UTR	COL4A1	28369186
13	110802678	C	A	3' UTR	COL4A1	27666438
13	110802679	C	A	3' UTR	COL4A1	27666438
15	23810779	GTCAG	G	promoter	MKRN3	29763903
15	23810849	C	T	promoter	MKRN3	30462148
15	35080829	A	G	3' UTR	ACTC1	27139165
17	10536907	C	T	splicing	MYH3	31491409
17	10559406	C	T	5' UTR	MYH3	31491409
17	41277375	T	A	5' UTR	BRCA1	30075112
17	61996359	G	T	promoter	GH1	27252485
17	70117348	G	A	5' UTR	SOX9	28546996
18	28683379	C	G	promoter	DSC2	27531918
19	2249105	CA	C	promoter	AMH	31238341
19	49468612	GG	CT	5' UTR	FTL	28636169
X	38202566	G	T	enhancer	OTC	29282796
X	38211793	T	G	5' UTR	OTC	29282796
X	38211808	G	A	5' UTR	OTC	29282796
X	38211811	A	G	5' UTR	OTC	29282796
X	38211834	C	T	5' UTR	OTC	29282796
X	38211835	C	T	5' UTR	OTC	29282796
X	38211844	C	A	5' UTR	OTC	29282796
X	70442966	C	CT		GJB1	28283593
X	70443186	G	T		GJB1	28283593
X	70444424	C	T	3' UTR	GJB1	29236290
X	85302634	G	A	promoter	CHM	28271586
X	85302634	G	T	promoter	CHM	28271586
X	138612871	AC	A	promoter	F9	27865967
X	147031110	T	C	3' UTR	FMR1	26554012

References

1. Brodtkorb E, Strand J, Backe PH, Lund AM, Bjørås M, Rootwelt T, et al. Four novel mutations identified in Norwegian patients result in intermittent maple syrup urine disease when combined with the R301C mutation. Molecular Genetics and Metabolism 2010;100(4):324 – 332. http://www.sciencedirect.com/science/article/pii/S1096719210001812.
2. Al-Shali K, Cao H, Knoers N, Hermus AR, Tack CJ, Hegele RA. A Single-Base Mutation in the Peroxisome Proliferator-Activated Receptor $\gamma 4$ Promoter Associated with Altered in Vitro Expression and Partial Lipodystrophy. The Journal of Clinical Endocrinology & Metabolism 2004 11;89(11):5655–5660. https://doi.org/10.1210/jc.2004-0280.
3. Funnell AW, Wilson M, Ballester B, Mak K, Burdach J, Magan N, et al. A CpG Mutational Hotspot in a ONECUT Binding Site Accounts for the Prevalent Variant of Hemophilia B Leyden. The American Journal of Human Genetics 2013;92(3):460 – 467. http://www.sciencedirect.com/science/article/pii/S0002929713000748.
4. Roessler E, Hu P, Hong SK, Srivastava K, Carrington B, Sood R, et al. Unique Alterations of an Ultraconserved Non-Coding Element in the 3'UTR of ZIC2 in Holoprosencephaly. PLOS ONE 2012 07;7(7):1–6. https://doi.org/10.1371/journal.pone.0039026.
5. Haghighi K, Chen G, Sato Y, Fan GC, He S, Kolokathis F, et al. A human phospholamban promoter polymorphism in dilated cardiomyopathy alters transcriptional regulation by glucocorticoids. Human Mutation 2008;29(5):640–647. https://onlinelibrary.wiley.com/doi/abs/10.1002/humu.20692.
6. Sabatelli M, Moncada A, Conte A, Lattante S, Marangi G, Luigetti M, et al. Mutations in the 3' untranslated region of FUS causing FUS overexpression are associated with amyotrophic lateral sclerosis. Human Molecular Genetics 2013 07;22(23):4748–4755. https://doi.org/10.1093/hmg/ddt328.
7. Fang Q, Chen S, Wang Y, Jiang S, Zhang R, Hu C, et al. Functional analyses of the mutation nt-128 T→G in the hepatocyte nuclear factor-1α promoter region in Chinese diabetes pedigrees. Diabetic Medicine 2012;29(11):1456–1464. https://onlinelibrary.wiley.com/doi/abs/10.1111/j.1464-5491.2012.03626.x.
