# Supplementary material for: parSMURF, a high-performance computing tool for the genome-wide detection of pathogenic variants
Source: Gigascience. 2020 May 23;9(5):giaa052. doi: 10.1093/gigascience/giaa052 (PMC7244787; doi:10.1093/gigascience/giaa052)
Supplement: giaa052_Response_to_Reviewer_Comments_Original_Submission [file giaa052_response_to_reviewer_comments_original_submission.pdf]

Author's Response To Reviewer Comments

Close

Paper: GIGA-D-19-00126
parSMURF, a High Performance Computing tool for the genome-wide detection of pathogenic variants
Alessandro Petrini; Marco Mesiti, Ph.D.; Max Schubach, Ph.D.; Marco Frasca, Ph.D.; Daniel Danis, Ph.D.; Matteo Re, Ph.D.; Giuliano Grossi, Ph.D.; Luca Cappelletti; Tiziana Castrignano', Ph.D.; Peter N. Robinson, Ph.D.; Giorgio Valentini, Ph.D.

Dear Hans Zauner,

According to your recommendations, we provided a thorough revision of our work. In particular we carefully considered the issues about potential overfitting, parameter optimization and other important concerns raised by the reviewers. A more detailed experimental analysis has been conducted along with a detailed discussion of our analysis and reported in the main manuscript and in the Supplementary document. New sections, tables and figures have been included in the revised version of the paper.

At high level, we modified the following sections and we added the following new sections in the main manuscript:
- We slightly shortened the Introduction and the first parts of the Methods section to avoid repetitions
- We largely modified “Improving predictions of pathogenic Mendelian variants”
- We added a completely new section “Prediction performances of parSMURF with an independent Mendelian test set”
- We added a completely new section “Assessment of the effect on prediction performance of the variants imbalance across regulatory regions.”
- We added a completely new section “Analysis of the hyper-parameters”
- We added or modified Tables 5, 6, 7 and 8.
- We added novel Supplementary Information including 7 novel tables and 6 novel figures.

The modified and novel parts in the main manuscript are highlighted in blue.
According to your suggestion we registered the software at SciCrunch.org with RRID: SCR_017560.

In the remaining part of this document we include a detailed point-by-point Response to Reviewers. Our responses start with R:

Thank you for your and reviewers’ work,

With best regards

Giorgio Valentini

Reviewer reports:

Reviewer #1:
This paper describes a parallelized extension of an existing method for classifying pathogenic non-coding mutations (hyperSMURF). The specific implementation choices are reasonable and indeed achieve the desired decrease in running time.

One issue needs a bit more motivation. The entire premise of this effort is based on the assumption that it is necessary to perform the training many times, thus necessitating that it is efficient. Where does the need to continuously retrain the models arise from? The input features hardly change, they are standard genomic annotations. Is it the addition of labels? If so it would be interesting to see some examples where the predictions have actually changed over time or over different training inputs.

R: Yes, you are right: the input features are fixed and the performance are evaluated with respect to a fixed set of annotated SNVs. The necessity of performing multiple training comes mainly from the model selection process: parSMURF is characterized by a large number of learning (hyper)-parameters and hence we need to run multiple combinations of its parameters in order to find the near-optimal ones for the specific task at hand. As explained in the paper we adopted both a “brute force” grid-search but also a more efficient Bayesian optimization algorithm to find the near-optimal parameters. In both cases we need to train several times the hyper-ensemble of learning machines, and this is in principle time consuming, but using parallel computation we can perform this in a relatively fast and efficient way. Moreover, by finely tuning the learning parameters we can obtain better results, as shown by the experimental results.

l39: "Nevertheless these imbalance-aware methods suffer from low scalability" It is unclear why sampling suffers from low scalability. Please expand on this point.

R: We mean that imbalance-aware methods, even if they take into account the imbalance that characterize several types of genomic data, are all conceived for processing data with single stand-alone machines. This could be reasonable for medium-size data sets, but when we have big genomic data we need to distribute data and computation across multiple machines. According to the suggestion of the reviewer in the revised version we accordingly modify the manuscript in the following way: “Nevertheless these imbalance-aware methods have been usually implemented with no or very limited use of parallel computation techniques, thus making problematic their application to the analysis of big genomic data.”

Reviewer #2: In the manuscript "parSMURF, a High Performance Computing tool for the genome-wide detection of pathogenic variants" Petrini and co-authors present parSMURF1 and parSMURFn, two parallel implementations for multi-core computers and for multi-node HPC cluster environments, respectively, of a previously published approach for the scoring of pathogenic non-coding variants, i.e the hyperSMURF method (Schubach et al Sci. Reports 2017). In contrast to the hyperSMURF sequential version, parSMURF makes computationally efficient the auto-tuning of the learning parameters through two alternative approaches: one based on exhaustive grid search and a second one based on Bayesian optimisation. Authors benchmark the computational cost and the accuracy of the trained models on 3 simulated and 2 real datasets of genetic variants.

Auto-tuning of learning parameters is an important step in machine learning approaches that can lead to a significant accuracy improvement. Such step is often unaffordable on big data sets because of limited computing resources or high complexity. Authors implemented a successful algorithmic strategy capable of parallelising the parameter optimisation of the hyperSMURF approach with substantial reduction of computing cost and improved performance in the evaluated settings. This is definitely a valuable resource for the community and makes possible new fields of application in genomics and big data problems with highly unbalanced classes. While promising, the characterization of the hyperSMURF approach would benefit from the following additional work:

MAJOR COMMENTS:

1- In the current form of the work, it can not be excluded the possibility that the different parameter optimisation options evaluated are merely translating into different overfitting degrees. To rule out such a possibility, it is necessary to evaluate how the performance of the different models generalise on fully independent test sets for both pathogenic non-coding variants and trait-associated variants.

R. To properly address the issue raised by the reviewer, we collected novel Mendelian pathogenic variants by adding 64 newly positive (pathogenic) non-coding variants manually annotated according to a comprehensive literature review. We included only those variations and publications judged to provide plausible evidence of pathogenicity. A total of 64 unique non-coding Mendelian disease-associated variants were identified (Supplementary Table 7).
As negatives we used common variants downloaded from NCBI (i.e. variants of germline origin and having a minor allele frequency (MAF) of >=0.01 in at least one major population, with at least two unrelated individuals having the minor allele, where major populations are those included in the 1K genome project). We selected only those variants that are non-coding using jannovar. The final number

of negatives has been randomly sampled in such a way that the ratio positives/negatives in the original mendelian data set and in the new mendelian data set used for validation is quite similar.
We trained hyperSMURF and parSMURF on all the original Mendelian data set and then we tested the resulting models on the unseen separated new Mendelian data set used for validation.
Results show that the models are able to obtain relatively high AUPRC results, especially when parSMURF is applied, showing that our models can nicely generalize using an independent data set.
Results have been added to the new subsection "Prediction performances of parSMURF with an independent test set"

2- In order to further characterise the potential overfitting that could result from parameter optimisation, it is recommended to monitor the relation between the training AUROC/AUPRC versus the testing AUROC/AUPRC (mean values across the 10 fold cross-validation in both cases). The idea here is to evaluate whether the improvements in training AUROC/AUPRC are disproportionate in comparison with the associated improvements on the testing AUROC/AUPRC.

R: In the revised version we reported in Table 7 the AUROC/AUPRC results across both the training and test sets of the 10 fold cross-validation and we added a comment stating that results on the training data are, as expected, better but comparable with those obtained with the test data. Moreover, the ratio between training and testing AUROC and AUPRC is approximately the same with hyperSMURF and parSMURF. These results show that performance improvement is not due to overfitting, but to a proper choice of the hyper-parameters well-fitted to the characteristics of the problem.

3- parSMURF is based on the same set of features than hyperSMURF and ReMM score (Smedley et al. 2016). Are the feature importance differences between parSMURF and hyperSMURF driving their performance differences? An analysis and discussion of this aspect is needed. In this direction, which is the Spearman correlation between parSMURF, hyperSMURF and ReMM scores for the different positive and the negative variants? Could any positive variant be highlighted as an example were parSMURF ranking was much superior than hyperSMURF's one and discuss why in terms of the underlying features?

R: In the revised version we measured Spearman correlation between both hyperSMURF and parSMURF scores with each of the 26 features used to train the hyper-ensemble. We found that hyperSMURF and parSMURF achieved basically the same Spearman correlation on each feature (the Pearson correlation between the vectors of Spearman correlations of hyperSMURF and parSMURF is about 0.98). Both hyperSMURF and parSMURF achieved the largest Spearman correlation coefficients for features related to the evolutionary conservation of the site (e.g. vertebrate, mammalian and primate PhyloP scores) and for some epigenomic features (histone acetylation, methylation and DNAse hypersensitivity).
Hence we cannot explain the better results of parSMURF on the basis of feature importance differences.
The better results of parSMURF are obtained mainly by its capacity of auto-tuning its learning hyperparameters and its capacity to find a model that better fits the data.
In Section "Improving predictions of pathogenic Mendelian variants" we discussed this issue and in Table S3 of Supplementary Information we provided the Spearman correlation between hyperSMURF and parSMURF scores and each of the 26 features used to train the hyperensembles.
Moreover in the same Section we added Table 5 which shows some examples of SNVs involved in Mendelian diseases significantly better ranked by parSMURF with respect to hyperSMURF. Finally, Table S7 in the Supplementary Information further expands the results shown in Table 6 by listing the same variants, along with their associated OMIM, Pub Med ID of the related publication, the gene and the type of non-coding region in which the variant is included.

4- Authors should report and discuss the optimal parameters retained among the hyper-parameter spaces (Table 4 and 6) associated to the models evaluated in Tables 5 and 6. Which hyper-parameters drove the performance improvements and why? This is an important aspect for future machine learning applications

R: In the revised version we reported the optimal parameters found by both the grid search and the Bayesian optimization search, and we interpreted the reasons for which such parameters lead to the best results, outlining that in particular that the undersampling ratio of negatives and the number of partitions (i.e. the number of ensembles used as base learners) play a key role. We added a new subsection ("Analysis of the hyper-parameters") just before the Conclusions, to discuss these items and we reported the best hyper-parameters found in Tables 1 and 2 of the Supplementary Information.

5- Authors state: "we compared the auto-tuned results only with those obtained with the default learning parameters of hyperSMURF, since our previous studies showed that hyperSMURF outperforms state-of-the-art methods, such as CADD [14], GWAVA [27], Eigen [19] and DeepSea [18] with both Mendelian diseases and GWAS hits data [29]" However, in a recent study (Caron et al. Genome Biology 2019) it was shown that NCBoost and ReMM can be considered the reference state-of-the-art methods in the case of non-coding variants associated to Mendelian diseases. Authors also claim that "hyperSMURF significantly improves the prediction performances of state-of-the-art machine learning methods". However, in order to claim so, NCBoost and ReMM (pre-computed scores) should have been included in the current benchmark regarding Mendelian non-coding variants. Nonetheless, in opinion of this referee, rather than the comparison with other state-of-the-art methods for the prediction of pathogenic variants (which requires thorough evaluation across multiple settings), the main interest of this work is to provide a proof-of-concept of the fact that auto-tunning of parameters may lead to better performances in a real genomic problem. In that sense, authors could well stick to a face-to-face comparison between hyperSMURF and parSMURF, although adapting their claims accordingly to the provided evidences.

R: We agree with the reviewer that the main interest of this work is to provide a proof-of-concept of the fact that auto-tuning of parameters may lead to better performances in a real genomic problem. For this reason in the revised version we did not claim that hyperSMURF/parSMURF significantly improves over other methods, and we focused more on the comparison between hyperSMURF and parSMURF.
In particular we removed from the paper the claims about the comparison with state-of-the-art methods, including the claims cited by the reviewer, and we focused more on the fact that auto-tuning of learning parameters may lead to better performances in a real genomic problem.

6- In the so-called "Mendelian" set in Table 2, the distribution of pathogenic variants across types of non-coding regions (5'UTR, promoter, etc..) is highly dissimilar from that of the negative variants, which is overrepresented by intronic and intergenic variants (Supplementary Table 1 from Schubach et al Sci. Reports 2017). In a recent study (Caron et al. Genome Biology 2019) it was shown that most state-of-the-art methods including ReMM are significantly biased across regulatory region types, with UTR'5 and promoter regions receiving more pathogenic scores than intronic and intergenic variants. The trend is observed for both pathogenic and non-pathogenic variants. Thus, the high imbalance between the region types in the "Mendelian set" is probably leading to inflated AUROC and AUPRC values and might be ultimately distorting the comparative benchmark between hyperSMURF and parSMURF. To avoid such confounding factor, it is recommended to match the negative and positive sets by regulatory region type, both for the Mendelian and the GWAS set (Table 2).

R: We agree with the reviewer that pathogenic variants across types of non-coding regions are unevenly distributed with regard to those of the negative variants and variant included in the Mendelian and GWAS datasets are not an exception to this remark. To show that, we divided pathogenic and neutral variants across 6 types of regulatory types (intronic, non coding exonic, intragenic, 3’ UTR, 5’ UTR and others) and evaluated the ratio between pathogenic and neutral variants for each category.
Supplementary Tables S4 and S5 show very high difference of ratios between positive and negative samples for both datasets, agreeing again with the reviewer that for the Mendelian dataset intronic and intergenic variants are overrepresented with regard to other regulatory region types.
To test how this issue might have impacted the performance improvement of parSMURF, we followed the advice of the reviewer and performed several tests on both datasets by rebalancing the negative classes of each regulatory region, so that no class is overrepresented.
An in-depth coverage of the test has been presented in the newly added sub-section “Assessment of the effect on prediction performance of the variants imbalance across regulatory regions.”, while Supplementary figures 5 and 6 report the results of the new tests.
The increase of performance of parSMURF with respect to hyperSMURF is not driven by the under- or over-representation of variants belonging to a particular region type, but by the parSMURF capacity of automatically fine-tuning the set of hyper-parameters, according to the given task at hand. Moreover we show that the improvement of parSMURF is maintained across different regulatory region types.

MINOR COMMENTS:

7- Introduction (which actually includes the 3 first paragraphs of the Methods section) could be overall shortened, trying to avoid repetition of the same ideas

R: In the revised version we shortened the Introduction and the first three paragraphs of the Methods section to avoid repetitions.

8- In the introduction three methods are highlighted for the increased performance achieved in the predictions of pathogenic variants in non-coding regions "by adopting imbalance-aware techniques", i.e. GWAVA, NCBoost and hyperSMURF. However, the ReMM score from Smedley et al. 2016 already implemented both oversampling and downsampling strategies and should be cited here.

R: We cited ReMM in the Introduction

9- Plots of the ROC and PRC curves as supplementary material would be welcome

R: We added ROC and PRC curves in the Supplementary Information (Supplementary figures 1,2,3 and 4).

10- English editing by a professional service is recommended.

R: We carefully edited English in the revised version.

Close
