# Supplementary material for: parSMURF, a high-performance computing tool for the genome-wide detection of pathogenic variants
Source: Gigascience. 2020 May 23;9(5):giaa052. doi: 10.1093/gigascience/giaa052 (PMC7244787; doi:10.1093/gigascience/giaa052)
Supplement: giaa052_Response_to_Reviewer_Comments_Revision_1 [file giaa052_response_to_reviewer_comments_revision_1.pdf]

Author's Response To Reviewer Comments

Close

Dear Hans Zauner,

According to your recommendations, we provided a second revision of our work. In particular, we rigorously provided an alternative experimental setup and analysis regarding the issue raised by the reviewer, carefully reconsidering the re-balancing strategy. A detailed explanation of the modified experimental setup, as well as new results have been added to the manuscript and to the Supplementary material. In detail:

- we modified the subsection “Assessment of the effect on prediction performance of the variants imbalance across regulatory regions” to accommodate the description and analysis of the suggested experimental setup.
- new results are graphically presented in Figures S5 and S6 in the Supplementary materials, substituting the older one.

The modified and novel parts in the main manuscript are highlighted in blue.
In the remaining part of this document we include a detailed point-by-point Response to Reviewers.

Thank you for your and reviewers’ work,

With best regards
Giorgio Valentini

Reviewer reports:

Reviewer #2:

This reviewer appreciates the efforts done by the authors, who provided a substantially improved version of the manuscript reflecting a much more mature state of the research work. Authors indeed satisfactorily addressed virtually all of the previous remarks and questions of this reviewer, except for question 6. This was related to the per-region imbalance of negative and positive samples in the Mendelian and GWAS dataset:

In these regards, in the revised version authors actually corrected the per-region imbalance in the training set, but unfortunately they kept the original imbalance for the testing set, which they did for the sake of comparability with the original setting. However, by doing so, the influence of the class imbalance in the testing results continue to be not controlled.

Not surprisingly, as authors noticed: "Forcing the same categorical composition during training leads to a reduced performance in all categories". This is of course expected, as the model trained in the balanced set could not merely use the region biases to predict the original unbalanced test set, which was seemingly the case in the initial setting.
Thus, such decrease should not be judged as a pitiful of the "balanced model", but on the contrary, as an indication that the "unbalanced" model was over-inflating performances in an equally unbalanced test set.

Therefore, it is important that authors report the testing results on the balanced test set of a model trained on an equally balanced set. In such a way, (i) AUPRC values will become comparable across genomic regions in Sup Figures 5 and 6 (which is not the case in their current form), and (ii) overall performance ("global" category in these figures) will not be distorted by potential genomic regions

imbalances.

As a corollary of the previous remarks, the authors' claim stating that "These results show that it is important to include as many examples as possible for training, since "naive" per-category balancing techniques lead to performance decay", can not be derived from the evidence currently provided.

R: We agree with the reviewer that by not rebalancing the test set we cannot infer any conclusion about the influence of the per-region imbalance on the predictions. For this reason, according to the reviewer suggestions, we corrected the per-region imbalance not only in the training set but also in the testing set and the experiments have been repeated with this new experimental setting.
Figures S5 and S6 in the Supplementary Information have been updated with the new AUPRC results, showing that parSMURF outperforms hyperSMURF also by “per region balancing” for both training and test set. The results are in general enhanced with respect to the original imbalanced setting.
We accordingly updated the section “Assessment of the effect on prediction performance of the variants imbalance across regulatory regions” to describe the new experimental set-up and comment the new results.

Close
