# Supplementary material for: parSMURF, a high-performance computing tool for the genome-wide detection of pathogenic variants
Source: Gigascience. 2020 May 23;9(5):giaa052. doi: 10.1093/gigascience/giaa052 (PMC7244787; doi:10.1093/gigascience/giaa052)
Supplement: giaa052_Response_to_Reviewer_Comments_Revision_2 [file giaa052_response_to_reviewer_comments_revision_2.pdf]

Author's Response To Reviewer Comments

Close

Dear Hans Zauner,

According to your recommendations, we provided the final revision of our work. According to your suggestions, we modified the manuscript for accomodating the final remarks. In detail:
- we got in contact with the GigaDB curators for providing the dataset used in the paper. Data will be uploaded to the GigaDB database shortly. A reference (56) to the upcoming GigaDB database has been added in the "Data Availability" section.
- DOI for our OSF data in reference 55 has been added
- coloring and text striking had been removed

Thank you for your and reviewers' work,

With best regards

Giorgio Valentini

Close
