# Supplementary material for: parSMURF, a high-performance computing tool for the genome-wide detection of pathogenic variants
Source: Gigascience. 2020 May 23;9(5):giaa052. doi: 10.1093/gigascience/giaa052 (PMC7244787; doi:10.1093/gigascience/giaa052)
Supplement: giaa052_Response_to_Reviewer_Comments_Revision_3 [file giaa052_response_to_reviewer_comments_revision_3.pdf]

Author's Response To Reviewer Comments

Close

I insert the doi for my new GigaDB dataset (http://dx.doi.org/10.5524/100743) in reference nr. 56 of the bibliography.

Close
