# Supplementary material for: parSMURF, a high-performance computing tool for the genome-wide detection of pathogenic variants
Source: Gigascience. 2020 May 23;9(5):giaa052. doi: 10.1093/gigascience/giaa052 (PMC7244787; doi:10.1093/gigascience/giaa052)
Supplement: giaa052_Reviewer_1_Report_Original_Submission — Maria Chikina -- 5/28/2019 Reviewed [file giaa052_reviewer_1_report_original_submission.pdf]

Reviewer Report

Title: parSMURF, a High Performance Computing tool for the genome-wide detection of pathogenic variants

Version: Original Submission **Date:** 5/28/2019

Reviewer name: Maria Chikina

Reviewer Comments to Author:

This paper describes a parallelized extension of an existing method for classifying pathogenic non-coding mutations (hyperSMURF). The specific implementation choices are reasonable and indeed achieve the desired decrease in running time.

One issue needs a bit more motivation. The entire premise of this effort is based on the assumption that it is necessary to perform the training many times, thus necessitating that it is efficient. Where does the need to continuously retrain the models arise from? The input features hardly change, they are standard genomic annotations. Is it the addition of labels? If so it would be interesting to see some examples where the predictions have actually changed over time or over different training inputs.

l39: "Nevertheless these imbalance-aware methods suffer from low scalability" It is unclear why sampling suffers from low scalability. Please expand on this point.

Level of Interest

Please indicate how interesting you found the manuscript: Choose an item.

Quality of Written English

Please indicate the quality of language in the manuscript: Choose an item.

Declaration of Competing Interests

Please complete a declaration of competing interests, considering the following questions:

- Have you in the past five years received reimbursements, fees, funding, or salary from an organisation that may in any way gain or lose financially from the publication of this manuscript, either now or in the future?
- Do you hold any stocks or shares in an organisation that may in any way gain or lose financially from the publication of this manuscript, either now or in the future?
- Do you hold or are you currently applying for any patents relating to the content of the manuscript?

- Have you received reimbursements, fees, funding, or salary from an organization that holds or has applied for patents relating to the content of the manuscript?
- Do you have any other financial competing interests?
- Do you have any non-financial competing interests in relation to this paper?

If you can answer no to all of the above, write 'I declare that I have no competing interests' below. If your reply is yes to any, please give details below.

I declare that I have no competing interests

I agree to the open peer review policy of the journal. I understand that my name will be included on my report to the authors and, if the manuscript is accepted for publication, my named report including any attachments I upload will be posted on the website along with the authors' responses. I agree for my report to be made available under an Open Access Creative Commons CC-BY license (http://creativecommons.org/licenses/by/4.0/). I understand that any comments which I do not wish to be included in my named report can be included as confidential comments to the editors, which will not be published.

Choose an item.

To further support our reviewers, we have joined with Publons, where you can gain additional credit to further highlight your hard work (see: https://publons.com/journal/530/gigascience). On publication of this paper, your review will be automatically added to Publons, you can then choose whether or not to claim your Publons credit. I understand this statement.

Yes Choose an item.
