# Supplementary material for: parSMURF, a high-performance computing tool for the genome-wide detection of pathogenic variants
Source: Gigascience. 2020 May 23;9(5):giaa052. doi: 10.1093/gigascience/giaa052 (PMC7244787; doi:10.1093/gigascience/giaa052)
Supplement: giaa052_Reviewer_2_Report_Original_Submission — Antonio Rausell -- 6/27/2019 Reviewed [file giaa052_reviewer_2_report_original_submission.pdf]

Reviewer Report

Title: parSMURF, a High Performance Computing tool for the genome-wide detection of pathogenic variants

Version: Original Submission **Date:** 6/27/2019

Reviewer name: Antonio Rausell

Reviewer Comments to Author:

In the manuscript "parSMURF, a High Performance Computing tool for the genome-wide detection of pathogenic variants" Petrini and co-authors present parSMURF1 and parSMURFn, two parallel implementations for multi-core computers and for multi-node HPC cluster environments, respectively, of a previously published approach for the scoring of pathogenic non-coding variants, i.e the hyperSMURF method (Schubach et al Sci. Reports 2017). In contrast to the hyperSMURF sequential version, parSMURF makes computationally efficient the auto-tuning of the learning parameters through two alternative approaches: one based on exhaustive grid search and a second one based on Bayesian optimisation. Authors benchmark the computational cost and the accuracy of the trained models on 3 simulated and 2 real datasets of genetic variants.

Auto-tuning of learning parameters is an important step in machine learning approaches that can lead to a significant accuracy improvement. Such step is often unaffordable on big data sets because of limited computing ressources or high complexity. Authors implemented a successful algorithmic strategy capable of parallelising the parameter optimisation of the hyperSMURF approach with substantial reduction of computing cost and improved performance in the evaluated settings. This is definitely a valuable ressource for the community and makes possible new fields of application in genomics and big data problems with highly umbalanced classes. While promising, the characterization of the hyperSMURF approach would benefit from the following additional work:

MAJOR COMMENTS:

1- In the current form of the work, it can not be excluded the possibility that the different parameter optimisation options evaluated are merely translating into different overfitting degrees. To rule out such possibility, it is necessary to evaluate how the performance of the different models generalise on fully independent test sets for both pathogenic non-coding variants and trait-associated variants.

2- In order to further characterise the potential overfitting that could result from parameter optimisation, it is recommended to monitor the relation between the training AUROC/AUPRC versus the testing AUROC/AUPRC (mean values across the 10 fold cross-validation in both cases). The idea here is to evaluate whether the improvements in training AUROC/AUPRC are disproportionate in comparison with the associated improvements on the testing AUROC/AUPRC.

3- parSMURF is based on the same set of features than hyperSMURF and ReMM score (Smedley et al. 2016). Are the feature importance differences between parSMURF and hyperSMURF driving their performance differences? An analysis and discussion of this aspect is needed. In this direction, which is the Spearman correlation between parSMURF, hyperSMURF and ReMM scores for the different positive and the negative variants? Could any positive variant be highlighted as an example were parSMURF

ranking was much superior than hyperSMURF's one and discuss why in terms of the underlying features?

4- Authors should report and discuss the optimal parameters retained among the hyper-parameter spaces (Table 4 and 6) associated to the models evaluated in Tables 5 and 6. Which hyper-parameters drove the performance improvements and why? This is an important aspect for future machine learning applications

5- Authors state: "we compared the auto-tuned results only with those obtained with the default learning parameters of hyperSMURF, since our previous studies showed that hyperSMURF outperforms state-of-the-art methods, such as CADD [14], GWAVA [27], Eigen [19] and DeepSea [18] with both Mendelian diseases and GWAS hits data [29]" However, in a recent study (Caron et al. Genome Biology 2019) it was shown that NCBoost and ReMM can be considered the reference state-of-the-art methods in the case of non-coding variants associated to Mendelian diseases. Authors also claim that "hyperSMURF significantly improves the prediction performances of state-of-the-art machine learning methods". However, in order to claim so, NCBoost and ReMM (pre-computed scores) should have been included in the current benchmark regarding Mendelian non-coding variants. Nonetheless, in opinion of this referee, rather than the comparison with other state-of-the-art methods for the prediction of pathogenic variants (which requires thorough evaluation across multiple settings), the main interest of this work is to provide a proof-of-concept of the fact that auto-tunning of parameters may lead to better performances in a real genomic problem. In that sense, authors could well stick to a face-to-face comparison between hyperSMURF and parSMURF, although adapting their claims accordingly to the provided evidences.

6- In the so-called "Mendelian" set in Table 2, the distribution of pathogenic variants across types of non-coding regions (5'UTR, promoter, etc..) is highly dissimilar from that of the negative variants, which is overrepresented by intronic and intergenic variants (Supplementary Table 1 from Schubach et al Sci. Reports 2017). In a recent study (Caron et al. Genome Biology 2019) it was shown that most state-of-the-art methods including ReMM are significantly biased across regulatory region types, with UTR'5 and promoter regions receiving more pathogenic scores than intronic and intergenic variants. The trend is observed for both pathogenic and non-pathogenic variants. Thus, the high imbalance between the region types in the "Mendelian set" is probably leading to inflated AUROC and AUPRC values and might be ultimately distorting the comparative benchmark between hyperSMURF and parSMURF. To avoid such confounding factor, it is recommended to match the negative and positive sets by regulatory region type, both for the Mendelian and the GWAS set (Table 2).

MINOR COMMENTS:

7- Introduction (which actually includes the 3 first paragraphs of the Methods section) could be overall shortened, trying to avoid repetition of the same ideas

8- In the introduction three methods are highlighted for the increased performance achieved in the predictions of pathogenic variants in non-coding regions "by adopting imbalance-aware techniques", i.e. GWAVA, NCBoost and hyperSMURF. However, the ReMM score from Smedley et al. 2016 already implemented both oversampling and downsampling strategies and should be cited here.

9- Plots of the ROC and PRC curves as supplementary material would be welcome

10- English editing by a professional service is recommended.

Level of Interest

Please indicate how interesting you found the manuscript: Choose an item.

Quality of Written English

Please indicate the quality of language in the manuscript: Choose an item.

Declaration of Competing Interests

Please complete a declaration of competing interests, considering the following questions:

- Have you in the past five years received reimbursements, fees, funding, or salary from an organisation that may in any way gain or lose financially from the publication of this manuscript, either now or in the future?
- Do you hold any stocks or shares in an organisation that may in any way gain or lose financially from the publication of this manuscript, either now or in the future?
- Do you hold or are you currently applying for any patents relating to the content of the manuscript?
- Have you received reimbursements, fees, funding, or salary from an organization that holds or has applied for patents relating to the content of the manuscript?
- Do you have any other financial competing interests?
- Do you have any non-financial competing interests in relation to this paper?

If you can answer no to all of the above, write 'I declare that I have no competing interests' below. If your reply is yes to any, please give details below.

I declare that I have no competing interests

I agree to the open peer review policy of the journal. I understand that my name will be included on my report to the authors and, if the manuscript is accepted for publication, my named report including any attachments I upload will be posted on the website along with the authors' responses. I agree for my report to be made available under an Open Access Creative Commons CC-BY license (http://creativecommons.org/licenses/by/4.0/). I understand that any comments which I do not wish to be included in my named report can be included as confidential comments to the editors, which will not be published.

Choose an item.

To further support our reviewers, we have joined with Publons, where you can gain additional credit to further highlight your hard work (see: https://publons.com/journal/530/gigascience). On publication of

this paper, your review will be automatically added to Publons, you can then choose whether or not to claim your Publons credit. I understand this statement.

Yes Choose an item.
