# Supplementary material for: parSMURF, a high-performance computing tool for the genome-wide detection of pathogenic variants
Source: Gigascience. 2020 May 23;9(5):giaa052. doi: 10.1093/gigascience/giaa052 (PMC7244787; doi:10.1093/gigascience/giaa052)
Supplement: giaa052_Reviewer_2_Report_Revision_1 — Antonio Rausell -- 1/21/2020 Reviewed [file giaa052_reviewer_2_report_revision_1.pdf]

Reviewer Report

Title: parSMURF, a High Performance Computing tool for the genome-wide detection of pathogenic variants

Version: Revision 1 **Date:** 1/21/2020

Reviewer name: Antonio Rausell

Reviewer Comments to Author:

This reviewer appreciates the efforts done by the authors, who provided a substantially improved version of the manuscript reflecting a much more mature state of the research work. Authors indeed satisfactorily addressed virtually all of the previous remarks and questions of this reviewer, except for question 6. This was related to the per-region imbalance of negative and positive samples in the Mendelian and GWAS dataset:
In these regards, in the revised version authors actually corrected the per-region imbalance in the training set, but unfortunately they kept the original imbalance for the testing set, which they did for the sake of comparability with the original setting. However, by doing so, the influence of the class imbalance in the testing results continue to be not controlled. Not surprisingly, as authors noticed: "Forcing the same categorical composition during training leads to a reduced performance in all 439 categories". This is of course expected, as the model trained in the balanced set could not merely use the region biases to predict the original unbalanced test set, which was seemingly the case in the initial setting.
Thus, such decrease should not be judged as a pitiful of the "balanced model", but on the contrary, as an indication that the "unbalanced" model was over-inflating performances in an equally unbalanced test set. Therefore, it is important that authors report the testing results on the balanced test set of a model trained on an equally balanced set. In such a way, (i) AUPRC values will become comparable across genomic regions in Sup Figures 5 and 6 (which is not the case in their current form), and (ii) overall performance ("global" category in these figures) will not be distorted by potential genomic regions imbalances.
As a corollary of the previous remarks, the authors' claim stating that "These results show that it is important to include as many examples as possible for training, since "naive" per-category 442 balancing techniques lead to performance decay", can not be derived from the evidence currently provided.

Level of Interest

Please indicate how interesting you found the manuscript: Choose an item.

Quality of Written English

Please indicate the quality of language in the manuscript: Choose an item.

Declaration of Competing Interests

Please complete a declaration of competing interests, considering the following questions:

- Have you in the past five years received reimbursements, fees, funding, or salary from an organisation that may in any way gain or lose financially from the publication of this manuscript, either now or in the future?
- Do you hold any stocks or shares in an organisation that may in any way gain or lose financially from the publication of this manuscript, either now or in the future?
- Do you hold or are you currently applying for any patents relating to the content of the manuscript?
- Have you received reimbursements, fees, funding, or salary from an organization that holds or has applied for patents relating to the content of the manuscript?
- Do you have any other financial competing interests?
- Do you have any non-financial competing interests in relation to this paper?

If you can answer no to all of the above, write 'I declare that I have no competing interests' below. If your reply is yes to any, please give details below.

I declare that I have no competing interests

I agree to the open peer review policy of the journal. I understand that my name will be included on my report to the authors and, if the manuscript is accepted for publication, my named report including any attachments I upload will be posted on the website along with the authors' responses. I agree for my report to be made available under an Open Access Creative Commons CC-BY license (http://creativecommons.org/licenses/by/4.0/). I understand that any comments which I do not wish to be included in my named report can be included as confidential comments to the editors, which will not be published.

Choose an item.

To further support our reviewers, we have joined with Publons, where you can gain additional credit to further highlight your hard work (see: https://publons.com/journal/530/gigascience). On publication of this paper, your review will be automatically added to Publons, you can then choose whether or not to claim your Publons credit. I understand this statement.

Yes Choose an item.
